# DMSOP-cleaving enzymes are diverse and widely distributed in marine microorganisms

**DOI:** 10.1038/s41564-023-01526-4

**Published:** 2023-11-29

**Authors:** Ornella Carrión, Chun-Yang Li, Ming Peng, Jinyan Wang, Georg Pohnert, Muhaiminatul Azizah, Xiao-Yu Zhu, Andrew R. J. Curson, Qing Wang, Keanu S. Walsham, Xiao-Hua Zhang, Serena Monaco, James M. Harvey, Xiu-Lan Chen, Chao Gao, Ning Wang, Xiu-Juan Wang, Peng Wang, Stephen J. Giovanonni, Chih-Ping Lee, Christopher P. Suffridge, Yu Zhang, Ziqi Luo, Dazhi Wang, Jonathan D. Todd, Yu-Zhong Zhang

**Affiliations:** 1 MOE Key Laboratory of Evolution and Marine Biodiversity, Frontiers Science Center for Deep Ocean Multispheres and Earth System & College of Marine Life Sciences, Ocean University of China, Qingdao, China; 2 School of Biological Sciences, University of East Anglia, Norwich, UK; 3 State Key Laboratory of Microbial Technology, Marine Biotechnology Research Center, Shandong University, Qingdao, China; 4 Institute of Inorganic and Analytical Chemistry, Bioorganic Analytics, Friedrich Schiller University Jena, Jena, Germany; 5 School of Pharmacy, University of East Anglia, Norwich, UK; 6 Department of Chemistry, King’s College London, London, UK; 7 Laboratory for Marine Biology and Biotechnology, Pilot National Laboratory for Marine Science and Technology (Qingdao), Qingdao, China; 8 Department of Microbiology, Oregon State University, Corvallis, OR USA; 9 State Key Laboratory of Marine Environmental Science/College of the Environment and Ecology, Xiamen University, Xiamen, China; 10 Frontiers Science Center for Deep Ocean Multispheres and Earth System, Qingdao, China; 11 Joint Research Center for Marine Microbial Science and Technology, Shandong University and Ocean University of China, Qingdao, China

**Keywords:** Biogeochemistry, Water microbiology

## Abstract

Dimethylsulfoxonium propionate (DMSOP) is a recently identified and abundant marine organosulfur compound with roles in oxidative stress protection, global carbon and sulfur cycling and, as shown here, potentially in osmotolerance. Microbial DMSOP cleavage yields dimethyl sulfoxide, a ubiquitous marine metabolite, and acrylate, but the enzymes responsible, and their environmental importance, were unknown. Here we report DMSOP cleavage mechanisms in diverse heterotrophic bacteria, fungi and phototrophic algae not previously known to have this activity, and highlight the unappreciated importance of this process in marine sediment environments. These diverse organisms, including *Roseobacter*, SAR11 bacteria and *Emiliania huxleyi*, utilized their dimethylsulfoniopropionate lyase ‘Ddd’ or ‘Alma’ enzymes to cleave DMSOP via similar catalytic mechanisms to those for dimethylsulfoniopropionate. Given the annual teragram predictions for DMSOP production and its prevalence in marine sediments, our results highlight that DMSOP cleavage is likely a globally significant process influencing carbon and sulfur fluxes and ecological interactions.

## Abstract (web summary)

Cycling of the sulfur compound DMSOP by dimethylsulfoniopropionate lyase enzymes in the most abundant marine bacteria, algae and fungi is diverse and prevalent in Earth’s oceans and sediments and probably impacts climate-active gas production.

## Main

**Fig. 1:**
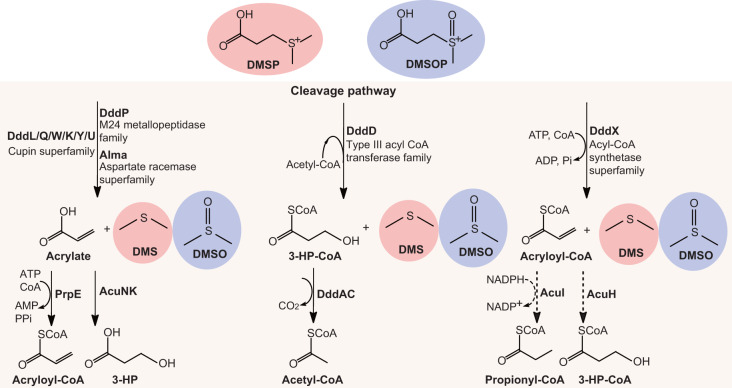
DMSP and DMSOP cleavage pathways. The three distinct pathways for DMSP and DMSOP cleavage are indicated, as are the key catabolic enzymes. DMSP and DMSOP-specific products are shown in pink and lilac shading, respectively. Dotted lines represent unconfirmed steps of the DddX DMSP cleavage pathway. 3-HP, 3-hydroxypropionate; ATP, adenosine triphosphate; ADP, adenosine diphosphate; AMP, adenosine monophosphate; Pi, inorganic phosphate; PPi, pyrophosphate; NADPH, nicotinamide adenine dinucleotide phosphate. Source data

Microorganisms in Earth’s oceans and marine sediments produce >10^9^ tons of the organosulfur compound dimethylsulfoniopropionate (DMSP) annually^1,2^ for its role as an anti-stress, storage and signalling compound^3–7^. DMSP is a major carbon and sulfur source for marine microorganisms^8,9^ via DMSP catabolic pathways that generate climate-active gases^10,11^, including methanethiol via bacterial DMSP demethylation^12^ or dimethylsulfide (DMS) via DMSP cleavage in algae, bacteria and fungi^13^ (Fig. 1). Recently, Thume et al.^14^ showed that many marine algae and bacteria oxidize DMSP to produce teragram quantities globally of the metabolite dimethylsulfoxonium propionate (DMSOP). DMSOP is thought to protect cells against oxidative stress^14,15^. Many diverse marine bacteria cleave DMSOP to yield dimethyl sulfoxide (DMSO) and a three-carbon co-product via unidentified DMSOP-cleaving enzymes, proposed to be independent of known ‘Ddd’ DMSP lyases (Fig. 1)^14^. Consequently, DMSOP production potentially limits the amounts of DMSP available for DMSP cleavage and, thus, generation of the climate-cooling gas^10,16^ and signalling molecule^17^ DMS in favour of DMSO, whose concentration often exceeds that of DMS(P)^18,19^. Without knowing the identity of the DMSOP-cleaving genes/enzymes it is impossible to comprehend the scale, diversity and importance of DMSOP cycling in marine organisms and environments. In this Article, we report DMSOP cleavage mechanisms in diverse heterotrophic bacteria, fungi and phototrophic algae, and highlight the unappreciated importance of DMSOP in marine sediment environments.

## Results

### DMSOP is abundant in saltmarsh sediments

DMSOP was previously detected at 0.14 ± 0.18 nM in seawater from major ocean basins^14^, but it has never been studied in marine sediments where DMSP can be three orders of magnitude more concentrated^20^. Importantly, we found that varied surface saltmarsh sediments contained total DMSOP levels ranging from 0.5 ± 0.1 mM to 3.4 ± 0.2 mM, which were orders of magnitude above the reported seawater levels^14^ and 2.6- to 13-fold higher than DMSP in these samples (Supplementary Fig. 1). Note, 46–72% of the sediment-associated DMSOP was in the particulate form (Supplementary Fig. 1). These data highlight saltmarsh sediments as niche environments for high DMSOP production/accumulation and that the previous reported teragram DMSOP sulfur flux^14^ was probably a significant underestimation.

### DMSOP is a potential osmoprotectant

Given that several bacteria import DMSOP^14^ and the abundance of DMSOP in marine sediments, we proposed that microorganisms could utilize it as an osmoprotectant, as is the case for DMSP^21,22^. To test this hypothesis, the osmosensitive *Escherichia* *coli* strain FF4169 (ref. ^23^) was grown under saline conditions in the presence and absence of DMSOP, DMSP and the nitrogenous osmoprotectant glycine betaine (GB). GB, DMSP and DMSOP significantly enhanced growth of FF4169 to similar levels in saline medium compared with control conditions lacking these zwitterionic compounds (Supplementary Fig. 2). These data demonstrated a potential role of DMSOP in osmotolerance, which had implications for DMSOP catabolism since organisms may not always want to readily degrade it. Recently, Azizah and Pohnert^15^ showed DMSOP accumulation in *Pelagibaca bermudensis* to be upregulated by oxidative stress and not increased by salinity, whereas DMSP levels, which were orders of magnitude higher than DMSOP, exhibited osmoregulatory patterns^15^. Thus, DMSOP may have different, for example, osmoregulatory or antioxidant, roles in different organisms that produce and/or accumulate it, as is the case for DMSP^2^.

### The DddY DMSP lyase also cleaves DMSOP to DMSO and acrylate

**Fig. 2:**
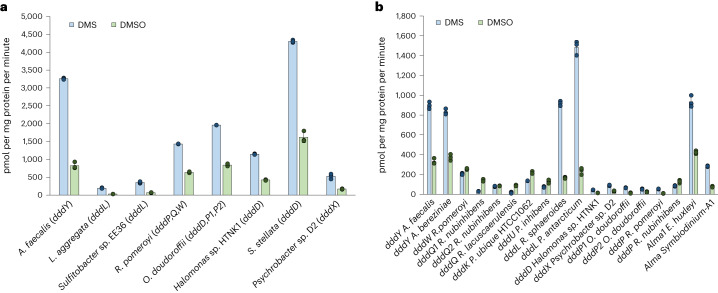
In vivo DMSP and DMSOP lyase assays with DMSP and DMSOP simultaneously added at equimolar levels. **a**, DMSP and DMSOP lyase activities from model organisms with known DMSP lyases. The complement of *ddd* genes found in tested bacteria are indicated in parentheses. **b**, DMSP and DMSOP lyase activities of Ddd and Alma enzymes expressed in *E. coli* BL21. Data are presented as mean ± s.d. (*n* = 3). Source data

**Fig. 3:**
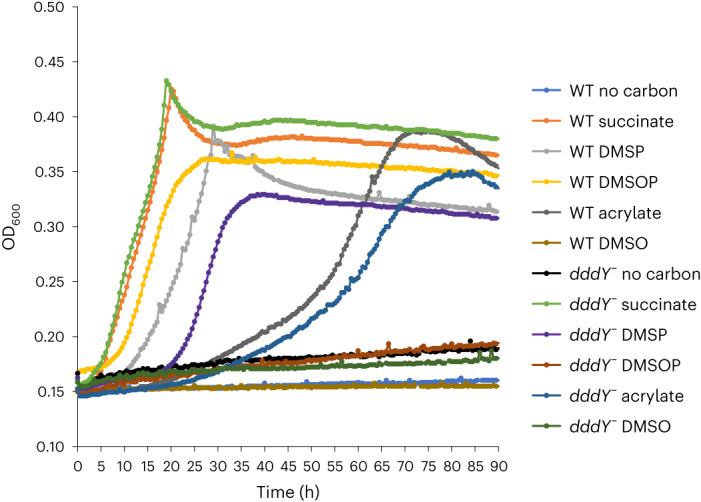
Growth curve of *A. faecalis* WT and *dddY*^−^ strains on succinate, DMSP, DMSOP, DMSO or acrylate as sole carbon source (2 mM). Growth curves with no carbon sources were performed as negative control. Data are presented as mean ± s.d. (*n* = 3).

We confirmed the previous report^14^ that *Alcaligenes faecalis*, which contains the DMSP lyase DddY, cleaved DMSOP to DMSO and acrylate (Supplementary Fig. 3) even with equimolar DMSP and DMSOP present (Fig. 2). This betaproteobacterium used DMSOP, like DMSP and acrylate^24^, as sole carbon source for growth producing 6.3 ± 0.8 mmol DMSO per mg protein after 90 h incubation (Fig. 3). Note, *A. faecalis* could not use DMSO as a carbon source (Fig. 3). Previously, wild-type (WT) *A. faecalis* and a *dddY*^*−*^ mutant strain were reported to have similar DMSOP lyase activities with 1 μM DMSOP added, implying that DddY lacked this activity and other unknown enzyme/s were responsible^14^. To identify such enzyme(s), an *A. faecalis* genomic library^24^ was screened for DMSOP lyase activity in *Rhizobium*, which cannot catabolize DMSOP. Two clones containing *dddY* conferred DMSOP lyase activity (200 ± 21.5 pmol DMSO per mg protein per minute; Supplementary Fig. 4). Cloned *dddY* from *A. faecalis* and *Acinetobacter bereziniae* conferred DMSOP lyase activity to *E. coli* (424.2 ± 20.6 and 359.4 ± 18.0 pmol DMSO per mg protein per minute, respectively) and purified DddY proteins yielded DMSO and acrylate from DMSOP (Supplementary Figs. 3 and 5). *A. faecalis* DddY had a *K*_m_ of 41.0 mM, a *k*_cat_ of 26.5 s^−^^1^ for DMSOP and displayed highest activities at 40 °C and pH 7.0 (Supplementary Figs. 6 and 7). The DddY *K*_m_ value for DMSOP was approximately five-fold higher than for DMSP (Supplementary Table 1) but was still in the millimolar range common for DMSP lyases^25–29^, which may be physiologically important as seen below. The DddY catalytic efficiencies, and in vitro (with purified DddY) and in vivo (with *E. coli* expressing DddY) experiments with equimolar DMSP and DMSOP levels, showed DddY to have 2.2–6.2-fold lower activity towards DMSOP than DMSP (Fig. 2, Supplementary Fig. 8 and Supplementary Table 1), consistent with DddY having a preference for DMSP over DMSOP.

In our hands, the *A. faecalis dddY*^*−*^ mutant grew on acrylate^24^, but not DMSOP, as the sole carbon source (Fig. 3) and showed 94% reduced DMSOP lyase activity compared with the WT when grown with 0.5 mM DMSOP (Supplementary Fig. 3). These data support DddY as the major *A. faecalis* DMSOP lyase. The 500-fold lower DMSOP levels used in Thume et al.^14^ probably explained why they saw no difference in DMSOP lyase activity between WT and *dddY*^*−*^ mutant strains, with the lower concentration unlikely to generate enough acrylate to induce *dddY* expression^24^. Note, there are probably other less significant unidentified DMSP/DMSOP lyases in *A. faecalis*, since DMSO and DMS production from DMSOP and DMSP, respectively, was not completely abolished in the *dddY*^*−*^ strain (Supplementary Fig. 3). Furthermore, the *dddY*^*−*^ mutant was able to grow on DMSP but not DMSOP after an extended incubation period^24^ (Fig. 3). Identification of the unknown DMSP and/or DMSOP lyase(s) is required to understand the mechanism(s) behind this growth phenotype.

### DMSOP cleavage is a universal trait of all known DMSP lyases

Ddd (spanning all nine enzymes) and Alma enzymatic activities on DMSP and DMSOP were examined in *E. coli*. As expected, all DMSP lyase genes conferred DMSP-dependent DMS production to *E. coli* (Supplementary Fig. 3). Like *dddY*, cloned *dddL*, *dddQ*, *dddW*, *dddK* and *dddU* conferred DMSOP lyase activity (Supplementary Fig. 3) even with equimolar DMSP present (Fig. 2), which was not surprising considering the similar structures of DMSP and DMSOP, and that these DMSP lyases have cupin domains and similar catalytic mechanisms^25,27–31^. Interestingly, the cupin DMSP lyases, especially most of the DddQ enzymes, conferred higher in vivo activities towards DMSOP than DMSP when both substrates were added at equimolar levels, except for DddL and DddY where the reverse was seen (Fig. 2). Significantly, *ddd* and *Alma* genes encoding the type III coenzyme A (CoA) transferase (DddD), acyl-CoA synthetase (DddX), M24 metallopeptidase (DddP), and the aspartate racemase (Alma) superfamily DMSP lyases from diverse bacteria, fungi and algae^26,32^ also showed DMSOP lyase activity (Supplementary Fig. 3) and with DMSP and DMSOP added at equimolar levels (Fig. 2). Of these non-cupin DMSP lyases, *Emiliania* *huxleyi Alma1* conferred the highest activity (381 ± 12.8 pmol DMSO per mg protein per minute), despite this bloom-forming coccolithophore, nor any other algae, being known to cleave DMSOP. *Psychrobacter dddX* also conferred high DMSOP lyase activity (279.7 ± 23.5 pmol DMSO per mg protein per minute), but the levels were much lower with *dddD* and *dddP* (Supplementary Fig. 3). Although most DMSP lyases cleaved DMSP and DMSOP at similar levels in the presence of both substrates, there were exceptions, for example, DddQ, DddL and Alma enzymes that may have evolved to differing degrees to favour DMSP or DMSOP. Note, there was variation in the preference of specific DddP and DddQ enzymes for DMSP or DMSOP in *E. coli*, for example, most DddP enzymes favoured DMSP but *Roseovarius nubinhibens* DddP showed a slight preference for DMSOP (Fig. 2), highlighting the need for careful functional analysis in these protein families.

Purified DddP, Alma and cupin-containing Ddd enzymes cleaved DMSOP into DMSO and acrylate (Supplementary Fig. 5). DddX also yielded DMSO from DMSOP but the co-product, thought to be acryloyl-CoA^26^, was not confidently identified (Supplementary Fig. 5). As with DddY, these enzymes exhibited millimolar *K*_m_ values for DMSOP ranging from 1 ± 0.2 mM (DddX) to 65 ± 10.9 mM (DddL), which were similar to those for DMSP^25–29,33,34^ (Supplementary Table 1, and Supplementary Figs. 7 and 9). These millimolar *K*_m_ values could effectively allow host organisms to accumulate DMSP/DMSOP at intracellular concentrations appropriate for physiological roles as an anti-stress compound, for example, in osmoprotection (Supplementary Fig. 2). Only the catalytic efficiency of DddW for DMSOP was similar to that for DMSP^25^ (Supplementary Table 1). In contrast, DddY, DddX, DddP, DddL and Alma were more efficient (3–13.3-fold) using DMSP than DMSOP, whereas DddK, DddQ and DddU were more efficient (2–83.4-fold) with DMSOP (Supplementary Table 1 and Supplementary Fig. 7). Note, DddP, DddX, DddY and Alma proteins generated 1.4–5-fold more DMS than DMSO when incubated with equimolar DMSP and DMSOP levels (Supplementary Fig. 8), consistent with them having significant DMSOP lyase activity but preferring DMSP as a substrate. In all cases the catalytic efficiency data conformed to the trends seen in the *E. coli* in vivo activity assays, but the magnitude of DMSP/DMSOP lyase activities differed between in vitro and in vivo assays (Fig. 2 and Supplementary Table 1). It is possible that assays with purified proteins did not give an accurate account of the enzymes working in their natural cellular environment. Nevertheless, all the diverse DMSP lyases should also be considered as DMSOP lyases. This has important environmental implications given the abundance and importance of the organisms containing these enzymes, for example, the abundant marine *Roseobacter*^35^, SAR11 bacteria^36,37^, ascomycete fungi and bloom-forming algae. However, given the disparity between some in vivo and in vitro data from *E. coli*, it was important to evaluate the performance of DMSP lyase enzymes on both DMSP and DMSOP in their natural hosts.

### Diverse marine bacteria, algae and fungi cleave DMSOP via their DMSP lyases

The ability of representative model organisms with known DMSP lyases to cleave DMSP and DMSOP was examined. As expected, all strains had DMSP lyase activity, including *Fusarium culmorum* with *dddP* (0.9 ± 0.1 pmol DMS per mg fresh weight per minute), *E. huxleyi* containing *Alma1* (Supplementary Fig. 10) and diverse bacteria (Supplementary Fig. 3). All tested bacterial strains also cleaved DMSOP at levels far above the control, *P. bermudensis*, which has no known DMSP lyases and no DMSP lyase activity. These strains included *Labrenzia aggregata* and *Sulfitobacter* sp. EE36 with *dddL* (2041.2 ± 46.1 and 586 ± 7.8 pmol DMSO per mg protein per minute); *Halomonas* sp. HTNK1, *Sagittula stellata* and *Oceanimonas doudoroffii*, all with *dddD* (ranging from 1,640 ± 24.9 to 1,712 ± 29.9 pmol DMSO per mg protein per minute); and *Psychrobacter* sp. D2 with *dddX* (1,663.6 ± 13.7 pmol DMSO per mg protein per minute; Supplementary Fig. 3). DMSOP lyase activities were reduced by ~97% in the *L. aggregata dddL*^*−*^ and *Halomonas* sp. HTNK1 *dddD*^*−*^ strains, and by 80% in the *Psychrobacter* sp. D2 *dddX*^*−*^ mutant (Supplementary Fig. 3), confirming that these DMSP lyases were the major drivers of DMSOP cleavage in these strains. Importantly, all bacteria showed significant DMSOP cleavage levels when incubated with both DMSOP and DMSP, but had higher DMSP lyase activity, consistent with DddY, DddL, DddD, DddP and DddX having a preference for DMSP and the *E. coli* work above (Fig. 2 and Supplementary Table 1).

The DMSP- and DMSOP-producing alga *E*. *huxleyi*^14^ (Supplementary Fig. 10) and plant pathogenic fungi *F. culmorum* (0.4 ± 0.04 pmol DMSO per mg fresh weight per minute) also cleaved DMSOP to DMSO, an activity not previously described in eukaryotes. *F. culmorum* showed six-fold higher lyase activity on DMSP than DMSOP when both substrates were present (0.6 ± 0.1 pmol DMS versus 0.1 ± 0.001 pmol DMSO per mg fresh weight per minute). *E. huxleyi* DMSP and DMSOP lyase activity was inhibited by Br–DMSP, a known Alma1 inhibitor^38^, indicating that DMSP/DMSOP cleavage was mediated by this enzyme (Supplementary Fig. 10). Note, *E. huxleyi* extracts also produced quantitatively more DMS than DMSO with both DMSP and DMSOP present, further indicating the preference of Alma1 for DMSP (Supplementary Fig. 10). These data confirm that the known DMSP lyases are robust indicators of both DMSP and DMSOP cleavage in diverse bacteria, algae and fungi; vastly extend the known range of organisms that cleave DMSOP^14^ and allow DMSOP cleavage potential to be investigated in environmental samples, as seen below.

### DMSOP is an important source of carbon and sulfur for abundant marine bacteria

**Fig. 4:**
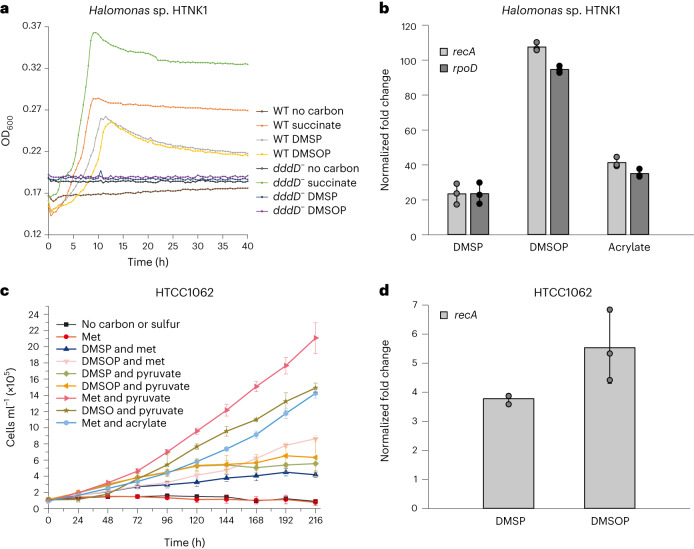
Analysis of DMSOP cleavage by key bacterial strains. **a**, Growth of *Halomonas* sp. HTNK1 WT and *dddD*^*−*^ strains on succinate, DMSP or DMSOP as sole carbon source (2 mM). **b**, RT–qPCR analysis of *Halomonas* sp. HTNK1 *dddD* in cells incubated with succinate (control) and succinate plus DMSP, DMSOP or acrylate (5 mM). Transcription of *dddD* was normalized to *recA* and *rpoD*. **c**, Growth curves of *P. ubique* HTCC1062 with 100 µM of pyruvate, acrylate, DMSP or DMSOP as carbon source and 25 µM of DMSP, DMSOP, DMSO or methionine (Met) as sulfur source. **d**, RT–qPCR analysis of *P. ubique* HTCC1062 *dddK* in cells grown with pyruvate and Met only (control) or amended with 100 µM DMSOP and DMSP (Methods). Expression of *dddK* was normalized to *recA*. Data are presented as mean ± s.d. (*n* = 3).

Some bacteria with Ddd enzymes, particularly those with DddD, DddX and DddY, utilize the DMSP cleavage three-carbon product as a carbon source and release DMS^24,26,39^ (Figs. 3 and 4). Indeed, *Oceanospirillales* *Halomonas* sp. HTNK1 grew similarly well with both DMSP and DMSOP as sole carbon sources, but the *dddD*^*−*^ strain no longer had this capacity (Fig. 4). Furthermore, *dddD* transcription was enhanced by DMSP and DMSOP substrates and acrylate, as previously reported^39^, but induction was higher (three- to five-fold) with DMSOP (Fig. 4).

The model SAR11 clade bacterium *Candidatus* Pelagibacter ubique HTCC1062 (with *dddK*)^40^ also used DMSP, DMSOP and their catabolites acrylate and 3-hydroxypropionate^40^ as carbon sources and MeSH^40^ and DMSO as sulfur sources (Fig. 4). It was noticeable that HTCC1062 grew better on DMSOP than on DMSP as the sole carbon source, consistent with DddK having a higher catalytic efficiency for DMSOP (Supplementary Table 1). Supporting this, DMSP and, to a greater extent, DMSOP induced *dddK* transcription (Fig. 4). Thus, representative strains of major groups of DMSP-degrading marine bacteria utilized DMSOP, like they did DMSP, as a carbon and/or sulfur source. Transcriptional induction of DMSP lyase genes by DMSOP/DMSP substrate and/or catabolites was probably key in organisms that used these compounds as a carbon source^24,39^.

### DMSP lyases have similar catalytic mechanisms for DMSP and DMSOP

**Fig. 5:**
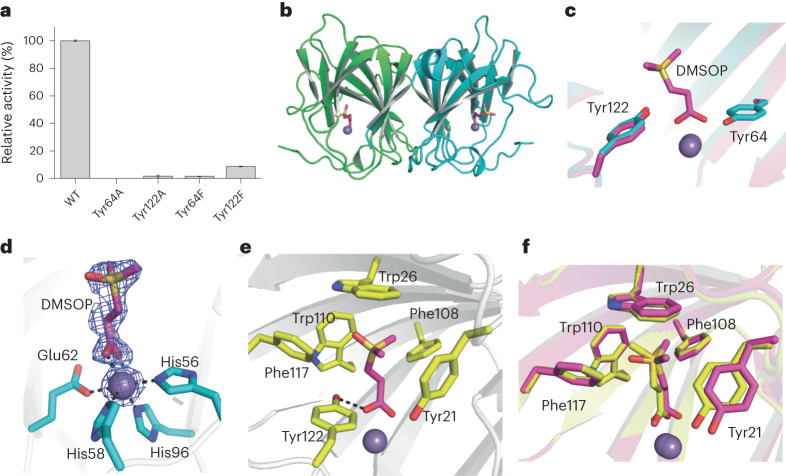
Structural and mutational analyses of DddK. **a**, DMSOP lyase activity of purified site-directed DddK mutant proteins where residues potentially involved in DMSOP catabolism were substituted as indicated. The enzymatic activity of WT DddK was defined as 100%. Results represent the mean of three independent experiments with error bars showing the respective s.d. **b**, Overall structure of the DddK–DMSOP complex. There are two DddK molecules arranged as a dimer in an asymmetric unit, which are coloured in green and cyan, respectively. The metal ion in DddK is shown as a purple sphere. The DMSOP molecule is shown in magenta sticks. **c**, Structural alignment of the DddK–DMSOP complex and WT DddK (PDB code: 6A53). The structure of DddK–DMSOP complex is shown in magenta, and the structure of WT DddK complex is shown in cyan. **d**, Residues and molecules involved in coordinating Mn^2+^ in DddK. The 2Fo-Fc densities for DMSOP and Mn^2+^ are contoured in blue meshes at 1.0*σ*. **e**, Residues involved in binding DMSOP. **f**, Structural alignment of important residues from DddK–DMSOP complex and DddK–DMSP complex (PDB code: 6A55). The structure of DddK–DMSOP complex is coloured in magenta, and the structure of DddK–DMSP complex in yellow.

We next investigated the DMSOP lyases catalytic mechanism(s). For SAR11 DddK, whose structure was previously solved (Protein Data Bank (PDB) code: 6A53)^29^, Tyr64 and Tyr122 were identified as potential catalytic residues^29,41^ and their substitution to alanine or phenylalanine abolished or exhibited >90% reduced DMSOP lyase activity, respectively (Fig. 5a). Circular dichroism spectroscopy analysis showed that these substitution mutants retained secondary structures similar to WT DddK (Supplementary Fig. 11), implying that Tyr64 was the catalytic residue for DMSOP cleavage, as it was for DMSP^29,41^, and that Tyr122 had a different but important role, as seen below.

We determined the crystal structure (1.62 Å) of the inactive Tyr64Ala DddK complexed with DMSOP (Fig. 5b and Supplementary Table 2), which aligned to structures of WT DddK and the DddK–DMSP complex (PDB code: 6A55), with root mean square deviations of 0.23 Å and 0.18 Å, respectively. Structural analysis also highlighted Tyr64 and Tyr122 as the probable DddK DMSOP cleavage catalytic residues (Fig. 5c), although the distance between the Tyr64 hydroxyl and the C-alpha of DMSOP was >4 Å. All DddK structures contained a metal ion, reported as Mn^2+^ (ref. ^29^), proposed to be important in DMSOP binding and catalysis^29^. Residues His56, His58, Glu62, His96 and the DMSOP molecule coordinated the Mn^2+^ (Fig. 5d). In addition to the coordination bond between Mn^2+^ and DMSOP, a hydrogen bond between Tyr122 and DMSOP helped locate the carboxyl group of DMSOP in the DddK active site, potentially explaining why the Tyr122 substitution reduced DMSOP lyase activity by >90% (Fig. 5e). The positively charged dimethylsulfoxonium moiety of DMSOP was located exactly where DMSP was in the DddK–DMSP complex and was stabilized via cation–π interactions to the side chains of several aromatic residues, including Tyr21, Trp26, Phe108, Trp110 and Phe117, which, except for Tyr21, also perfectly superposed onto the DddK–DMSP complex (Fig. 5f). The Tyr21 side chain moved ~1 Å away from DMSOP compared with that of DMSP (Fig. 5f), providing a wider substrate-binding pocket to accommodate the DMSOP dimethylsulfoxonium moiety, which is larger than the sulfonium of DMSP.

From these data, we proposed that the DddK catalytic mechanism for DMSOP mirrored that for DMSP^29^ (Supplementary Fig. 12). Before DMSOP enters the active site, His56, His58, Glu62 and His96 residues and a water molecule coordinate Mn^2+^ (ref. ^29^) (Supplementary Fig. 12a). Tyr64 forms a hydrogen bond with the water molecule activated by Mn^2+^, which may help the deprotonation of Tyr64 to act as a catalytic base^29^. When DMSOP binds to DddK, it displaces the water molecule and forms a new coordination bond with Mn^2+^ (Supplementary Fig. 12b). Subsequently, the catalytic residue Tyr64 attacks the C_α_–H proton of DMSOP, forming a C_α_ carbanion. Then, the C_α_ carbanion attacks the C_β_ of DMSOP, leading to the breaking of the C_β_–S bond (Supplementary Fig. 12c). Consequently, DMSOP is cleaved into DMSO and acrylate, which is then released from the DddK active site (Supplementary Fig. 12d).

We also solved the crystal structure of the DddY–DMSOP complex (Supplementary Table 2) and docked DMSOP into DddQ and DddP structures. In these structures, DMSOP located in the same position as acrylate/DMSP in DddY/DddQ/DddP complexes^27,28,42^ (Supplementary Figs. 13 and 14), suggesting that all known DMSP lyases adopt similar catalytic mechanisms to cleave both DMSP and DMSOP.

### Environmental importance of DMSOP cycling

**Fig. 6:**
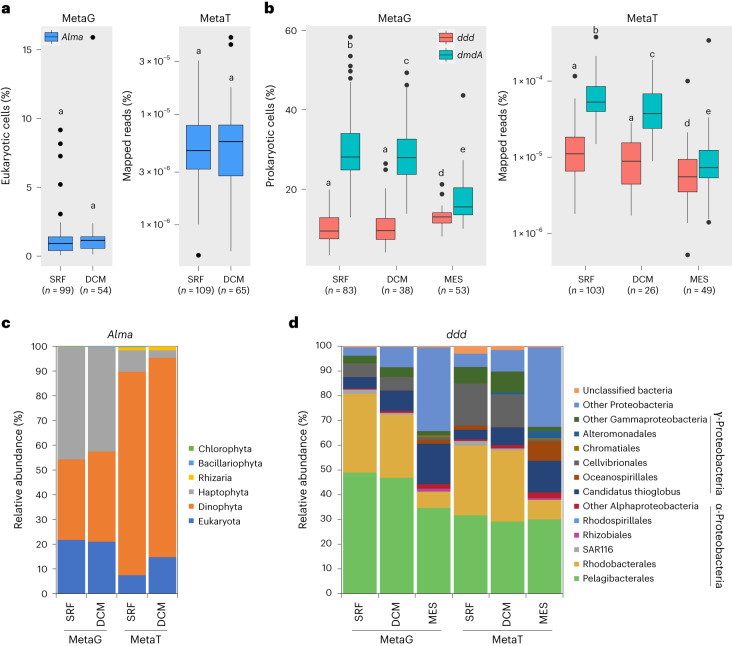
Distribution of genes encoding prokaryotic and eukaryotic DMSP lyases in Tara Oceans datasets OM-RGC-v2 (0.22–3 μm) and MATOU (0.8–20 μm), respectively. **a**, Relative abundance of eukaryotic *Alma* genes in 153 of 174 MATOU metagenomes and 174 of 176 metatranscriptomes from SRF water layers (0–10 m) and DCM layers (10–200 m). **b**, Relative abundance of the nine prokaryotic *ddd* genes and the DMSP demethylation gene *dmdA* in OM-RGC-v2 metagenomes and metatranscriptomes from SRF, DCM and MES water layers (200–1,000 m). **c**, Taxonomic assignment of *Alma* genes in the MATOU dataset. **d**, Taxonomic assignment of *ddd* genes in the OM-RGC-v2 dataset. Letters denote genes or transcripts that are significantly different (*P* < 0.05) between water layers determined by two-sided Wilcoxon test; a shared letter means no significant differences. Boxplots show median (centre line), upper and lower quartiles (box limits), the interquartile range (whiskers) and outliers (black dots). MetaG, metagenome; MetaT, metatranscriptome.

We estimated the relative abundance of *Alma* and *ddd* genes and their transcripts in Earth’s oceans by analysing Tara Ocean datasets (Fig. 6 and Supplementary Table 3), as in Landa et al.^43^ and Vorobev et al.^44^. Approximately 1.2% of eukaryotes, mostly dinoflagellates and haptophytes, in almost all surface (SRF) and the deep chlorophyll maximum (DCM) water samples were predicted to contain and express *Alma* genes at relatively low levels (Fig. 6 and Supplementary Table 3). A previous study covering fewer Tara Ocean stations but more size fractions showed that *Alma* transcripts were mostly detected in the 0.8–5 μm fraction in both SRF and DCM layers^44^. In contrast, the bacterial *ddd* genes, particularly *dddP* accounting for ~65% of total *ddd* genes, were cumulatively very abundant (present in 10–13% of marine prokaryotes in the SRF, DCM and mesopelagic (MES) layers) and transcribed in all samples (Fig. 6 and Supplementary Table 3). Despite a significant increase of gene relative abundance in MES samples compared with SRF and DCM waters, *ddd* genes showed decreased relative expression with depth (Fig. 6 and Supplementary Table 3), implying that DMSOP/DMSP cleavage is potentially more important in SRF seawaters (Supplementary Table 3). Note, *Roseobacter* and SAR11 *ddd* genes/transcripts dominated in SRF and DCM layers (Fig. 6). In MES samples, SAR11 *ddd* genes were still very abundant, whereas *Roseobacter* *ddd* genes vastly decreased, largely in favour of increased proteobacterial (in metagenomes) and *Oceanospirillales* (in metatranscriptomes) *ddd* genes (Fig. 6). Consistent with Landa et al.^43^, the *ddd* genes and transcripts were much less abundant in SRF, DCM and MES waters compared with the ubiquitous DMSP demethylation gene *dmdA* (Fig. 6 and Supplementary Table 3). Given the huge potential for marine DMSP demethylation, it should be a future priority to establish whether DmdA could demethylate DMSOP.

With surface saltmarsh sediments containing high total DMSOP levels (Supplementary Fig. 1), it was significant that ~13.5% of bacteria in such sediments were predicted to contain a *ddd* gene^20^. More diverse marine surface sediments were also previously highlighted as hotspots for DMSP accumulation and bacterial DMSP catabolic genes, particularly *dddP* (predicted in 4.0–15.6% surface marine sediment bacteria)^45,46^. It is possible that marine sediments in general are also rich in DMSOP and its microbial cycling, but further work is required to establish this. Given the previously predicted teragram DMSOP production budget^14^, that DMSOP is potentially abundant in sediments, the vast genetic potential for DMSOP catabolism in diverse seawater and marine sediments, and that representatives of the major groups of marine bacteria with this potential cleave DMSOP, DMSOP likely constitutes an important source of reduced carbon and sulfur in marine sediments. Thus, the importance of DMSOP as a marine nutrient is probably far less significant than for DMSP in seawater, given DMSOP was seen at 0.1–1% the levels of DMSP in most tested marine organisms and environments^14^. However, there were exceptions, for example, in the haptophyte *Isochrysis galbana* under stationary phase, where DMSOP reached ~75% of the DMSP levels^14^. In contrast, it is possible that DMSOP cycling may be equally or more important than DMSP cycling in marine sediments, where DMSOP levels can surpass DMSP. Note, standing stock concentrations may not accurately report metabolism and future work should consider both DMSP and DMSOP synthesis and catabolic rates.

## Discussion

Before this study, the scale, mechanism(s) and importance of DMSOP cycling in organisms and marine environments were unknown. We found DMSOP at millimolar levels in saltmarsh sediments, which were uniquely higher than DMSP and far more abundant than the 0.14 nM average reported seawater values^14^. These data highlight surface marine sediments, which contain far higher cell densities than seawater^20^, as potential niches for DMSOP production. Thus, the predicted teragram budget for DMSOP^14^ was probably vastly underestimated. Above this, a potential role for DMSOP in osmoregulation was elucidated. The role of DMSOP in organisms that accumulate it will probably depend on its concentration, cellular location and catabolism in the host, like DMSP^2^. To be a major osmolyte, DMSOP would have to accumulate to high intracellular concentrations, which is rare in known DMSOP producers^14^, and the ~500-fold higher DMSP seawater concentration over DMSOP would largely favour the former as being imported for osmoregulation. Indeed, DMSOP probably has an antioxidant role in the pelagic DMSOP-producing bacterium *P. bermudensis*^15^. DMSOP may be more commonly used for osmoregulation in marine sediments where DMSOP was more abundant than DMSP.

This study vastly extended the magnitude and biodiversity of DMSOP cleavage, from previously being confined to some marine bacteria, to being present in the most abundant marine bacterial groups and other domains of life, namely bloom-forming algae and pathogenic fungi. We elucidated exactly how these organisms cleave DMSOP, which is via their DMSP lyase enzymes that had varied catalytic efficiencies but similar mechanisms for DMSP and DMSOP cleavage. Moreover, clarification was provided on the potential importance of DMSOP, with DMSP/DMSOP lyase genes being very abundant (in 10–13% of marine prokaryotes) and transcribed in Earth’s marine waters and sediments, particularly from *Roseobacter* and SAR11, which together can account for ~45% of marine bacteria^35–37^ and who could use DMSOP as a carbon and sulfur source.

Ultimately, this work highlights DMSOP cleavage as a potentially important cog in marine and global sulfur and nutrient cycling, and as a major source of DMSO. It also challenges future studies to gain vital knowledge on the range of DMSOP-producing organisms, their DMSOP synthesis mechanism(s) and the environmental levels of DMSOP, unknown factors at large that are required to fully comprehend the global significance of this recently discovered organosulfur compound.

## Methods

### Chemical syntheses

DMSP was synthesized from DMS (Merck; 528021) and acrylic acid (Fisher Scientific; 164252500), as in Todd et al.^39^. DMSOP was synthesized from DMSP, as in Thume et al.^14^. Purity of DMSOP was analysed by nuclear magnetic resonance (NMR) spectroscopy using an Avance III HD Nanobay 400 mHz NMR spectrometer (Bruker). No traces of DMSO or DMSP were detected.

### DMSOP, DMSP, DMSO and GB concentrations in saltmarsh sediments analysed by NMR

Triplicate surface sediment samples (the upper 2 cm) from four saltmarshes in Norfolk, UK (Supplementary Table 4) were taken for DMSP, DMSOP, DMSO and GB analysis. To measure total concentrations, 0.5 g of sediments were diluted in 0.8 ml of D_2_O water (Fisher Scientific; 10255880), heated at 100 °C for 20 min to inactivate DMSP lyases and then allowed to cool. Heat-killed samples were then homogenized using a FastPrep-24 5 g bead beater (MP Biomedicals) for three cycles of 60 s at 6.0 m s^−^^1^. Samples were spun down and supernatants used for NMR analysis. For dissolved DMSOP, 0.5 g of sediment were diluted in 0.8 ml of D_2_O water, vortexed for 30 s and centrifuged. Supernatants were heat killed as above and analysed by NMR.

Subsequently, 5 µl of pyrazine 50 mM (Merck; 807064) was added to 495 µl of supernatants as internal standard and transferred to 5 mm NMR tubes. All NMR experiments were performed at 298 K on a Bruker 500 MHz spectrometer. The pulse sequence incorporated a double echo excitation sculpting component for water suppression (Bruker library zgesgp). Each sample was run at 256 scans and 1 s relaxation delay d1.

All spectra were phased, base-corrected and calibrated for the pyrazine peak at 8.63975 ppm. The chemical shifts of the GB, DMSP, DMSOP and DMSO diagnostic groups were ((CH_3_)_3_N) at 3.256 ppm, ((CH_3_)_2_S) at 2.913 ppm, ((CH_3_)_2_S) at 3.746 ppm and ((CH_3_)2 _S_) at 2.719 ppm, respectively, at 298 K.

GB, DMSP, DMSOP and DMSO final concentrations were obtained by calculating the ratio of the absolute integral of pyrazine (accounting for four protons) with the diagnostic peaks of GB (accounting for nine protons), DMSP, DMSOP and DMSO (accounting for six protons); these ratios were then multiplied by the dilution factor and the correction factor from the calibration curves. Calibration curve correction factors were 2.963, 2.719, 3.503 and 2.753 for GB, DMSP, DMSOP and DMSO, respectively. Calibration curves for all analytes were performed using 0.2–1.6 mM concentrations and 1 mM pyrazine. For each sample, a zgesgp at d1 = 1 s was recorded, and the data were plotted to obtain the correction factor. The detection limits for GB, DMSP, DMSOP and DMSO were 10, 15, 50 and 15 μM, respectively.

### Bacterial strains and growth conditions

Strains used in this study are shown in Supplementary Table 5. *A. faecalis* and *E. coli* strains were incubated in lysogeny broth (LB) (complete) or M9 (minimal) media^47^. *Rhizobium* *leguminosarum* was grown in TY (complete) or Y (minimal)^48^ media with 10 mM succinate. *L. aggregata*, *Sulfitobacter* sp. EE36, *Ruegeria pomeroyi*, *O. doudoroffii*, *Halomonas* sp. HTNK1, *S. stellata* and *P. bermudensis* were grown in YTSS (complete)^49^ or Marine Basal Medium (MBM; minimal)^50^ with 10 mM succinate as carbon source, except for *S. stellata*, for which 10 mM pyruvate was used. MBM salinity was adjusted to 35 practical salinity units (PSU) with sea salts (Merck; S9883). *Psychrobacter* sp. D2 was cultured in Marine broth 2216 (Merck; 76448) or M9 with 10 mM pyruvate. All strains were incubated at 30 °C, except for *E. coli* (37 °C) and *Psychrobacter* sp. D2 (25 °C).

*E. huxleyi* RCC173/CCMP373 was obtained from the Roscoff Culture Collection and cultured in K/2(-Tris -Si) medium^51^. Cultures were grown in a 14:10 h light:dark cycle with light provided by osram biolux lamps (40 µmol m^−2^ s^−1^ between 400 and 700 nm) at 18 °C to late exponential phase before assaying for DMSP and DMSOP lyase activities (as seen below).

### Sole carbon and sulfur source growth tests

*A. faecalis* and *Halomonas* sp. HTNK1 WT and mutant strains were grown overnight (16 h) in their respective complete media (as seen above). Then, optical density (OD)_600_ was adjusted to 0.6 and cells were washed three times with minimal media without carbon sources. Washed cells were inoculated into minimal media containing 2 mM DMSP, DMSOP, DMSO or acrylate. Succinate (2 mM) was used as positive control and media with no carbon source were used as negative control. Growth curves were performed in a SpectraMax iD5 microplate reader (Molecular Devices) at 30 °C, with readings taken at OD_600_.

*P. ubique* HTCC1062 was cultured as previously reported^52,53^. Briefly, HTCC1062 was grown in artificial seawater containing 100 μM carbon source (pyruvate, DMSP, acrylate or DMSOP), 25 μM glycine, 25 μM sulfur source (Met, DMSP, DMSOP or DMSO) and 1× vitamin mix2 at 18 °C. Growth of *P. ubique* HTCC1062 was monitored by flow cytometry using a CytoFlex S flow cytometer (Beckman Coulter).

All growth experiments were performed in triplicate.

### Osmoprotection experiments

Trehalose-deficient *E. coli* strain FF4169 (*otsA*^−^)^23^ was grown in LB medium and adjusted to OD_600_ of 0.3. Cells were washed twice with M63 minimal medium^54^ and inoculated into fresh M63 medium containing 22 mM glucose, 0.5 M NaCl and 1 mM DMSP, DMSOP or GB. Growth curves were performed at 37 °C in a Multiskan GO microplate reader (Fisher Scientific) with readings taken at OD_600_.

### Quantification of DMS and DMSO by gas chromatography

For DMSP-dependent DMS production, marine bacterial strains were grown in complete media overnight (16 h) and adjusted to an OD_600_ of 0.6. Cells were washed and diluted 1:10 into 2 ml sealed vials containing 0.3 ml of M9 or MBM 35 PSU with 0.5 mM DMSP. Vials were incubated overnight (16 h) at 30 °C or 25 °C (*Psychrobacter* sp. D2) before measuring DMS in the headspace by gas chromatography (GC).

For DMSOP lyase activity, marine isolates were grown in complete media as above and adjusted to an OD_600_ of 0.6. Cells were then washed and diluted 1:10 in 5 ml M9 or MBM 35 PSU with 0.5 mM DMSOP. After overnight (16 h) incubation at 30 °C or 25 °C (*Psychrobacter* sp. D2), 0.2 ml of cultures was aliquoted into 2 ml GC vials. Vials were then heated at 80 °C for 10 min to remove any possible DMS present in the cultures. Vials were left to cool down before adding 0.1 ml of 1 M Tin(II) chloride (SnCl_2_; Merck; 208256). Then vials were immediately sealed and incubated at 55 °C for 90 min to reduce DMSO and capture DMS, as in Lidbury et al.^55^. Vials were then left in the dark with shaking for 6 h at room temperature to allow equilibration of DMS between the liquid phase and the headspace before GC analysis. No DMS or DMSO was detected in heated control vials without SnCl_2_ or DMSOP added.

*E. coli* BL21(DE3) cells transformed with the plasmids described in Supplementary Table 6 were assayed with 0.1 mM isopropyl β-d-1-thiogalactopyranoside (Fisher Scientific; 10397642) and 0.5 mM DMSP or DMSOP, as in Carrión et al.^56^.

For DMSP and DMSOP lyase competition experiments, bacterial strains were inoculated into minimal media containing both DMSP and DMSOP at 0.5 mM concentration as described above. After 4 h incubation, samples were inactivated by heating at 90 °C for 15 min and cooled to room temperature, and the DMS generated from DMSP was quantified by GC. Thereafter, vials were opened and heated at 80 °C for 10 min to evaporate DMS in the cultures. Once cooled, 0.1 ml of 1 M SnCl_2_ was added to the vials, which were then immediately sealed. Subsequently, vials were incubated at 55 °C for 90 min to allow DMSO reduction and resultant DMS was left to equilibrate between the liquid phase and the headspace before GC analysis as above. Vials heated without SnCl_2_ added were included as controls to account for possible DMS remaining in the samples.

*Fusarium culmorum* Fu42 was grown on Potato Dextrose^57^ at 28 °C by Professor Paul Nicholson (John Innes Centre). Mycelial plugs from the growing edge (~10 mg) were inoculated into Y medium^48^ with 10 mM succinate, 0.5 mM DMSP or DMSOP or both substrates and 1 µg ml^−^^1^ of yeast extract. Vials were then sealed and incubated overnight (16 h) at 25 °C before measuring DMS and DMSO content by GC as described above. Amounts of DMS and DMSO produced were normalized by the milligram of fresh weight in each vial.

DMS generated from DMSP and the reduction of DMSO was quantified by GC using a flame photometric detector (Agilent 7890A GC fitted with a 7693 autosampler) and a HP-INNOWax 30 m × 0.320 mm capillary column (Agilent Technologies J&W Scientific). An eight-point calibration curve of DMS and DMSO standards was used and the detection limit for both compounds was 0.015 nmol. DMS and DMSO production rates are expressed as pmol per mg protein per minute and represent the mean of three biological replicates with their respective standard deviations (s.d.). Cellular protein content was estimated by a Bradford method, as in Carrión et al.^56^.

### Screening of *A*. *faecalis* genomic library

A genomic library of *A. faecalis*^24^ was transferred to *R. leguminosarum* J391 by triparental conjugation with an *E. coli* helper strain containing the plasmid pRK2013 (ref. ^58^). A total of 500 transconjugants were inoculated into Y medium^48^ with 5 mM DMSOP and incubated for 48 h at 30 °C before measuring the DMSO generated, as described above.

### GC–high-resolution mass spectrometry

*E. huxleyi* RCC173/CCMP373 cultures (50 ml each, 9.5 × 10^5^ cells ml^−^^1^) were centrifuged at 3,170*g*, concentrated into 1 ml and transferred into 4 ml vials with polytetrafluoroethylene/silicone septa. Cells were disrupted by sonication using nine cycles, 10-s pulses with 40% intensity with a Sonoplus ultrasound homogenizer (Bandelin). After adding ^13^C_2_–DMSOP and ^2^H_6_–DMSP to a 100 μM final concentration, vials were sealed and incubated for 20 min. Medium with no cells was used as abiotic control. Conversion of ^13^C_2_–DMSOP to ^13^C_2_–DMSO in samples was measured after reduction to ^13^C_2_–DMS by TiCl_3_, as previously described^14,59^. Conversion of ^2^H_6_–DMSP to ^2^H_6_–DMS in the samples was also determined. For each sample, a 1 ml aliquot was mixed with 200 µl 20% w/v TiCl_3_ (EMD Chemicals; 39562). Samples were heated at 55 °C for 1 h to allow reaction. All experiments were done in triplicate. To confirm Alma1’s role in DMSOP cleavage by *E. huxleyi*, the experiment above was repeated in triplicate with 50 μM Br–DMSP.

DMS extraction was achieved by immersing a solid phase microextraction fibre (50/30 µm DVB/CAR/PDMS, Supelco) in the samples’ headspace for 15 min at 20 °C, before GC analysis. DMS was desorbed into the S/SL injector at 250 °C (TRACE 1310, Thermo Scientific) fitted with a 30 m × 0.25 mm, 0.25 µm film ZB-1MS capillary column (Phenomenex). A hybrid quadrupole-Orbitrap mass spectrometer (Q-Exactive, Thermo Scientific) was used for detection. Ultrahigh-purity helium was used as carrier gas at a flow rate of 1.2 ml min^−1^. The oven temperature was held at 40 °C for 1 min, increased to 150 °C (15 °C min^−1^) and again held for 3.5 min. The transfer line and ion source were set to 250 °C and 300 °C, respectively. Mass measurements were performed in electron ionization-positive mode. A mass range from 45 to 200 *m*/*z* at 60,000 resolution was recorded. The ionization energy was 70 eV and scan time was 0.25 s. Data analyses were performed with the Thermo Xcalibur software v4.5.445.18 (Thermo Scientific; OPTON-30965).

The molecular ion traces of DMS (^12^C_2_H_6_^32^S), ^12^C_2_H_6_^34^S, ^13^C_2_H_6_^32^S and ^12^C_2_^2^H_6_^32^S were *m*/*z* 62.01845 ± 5 ppm, *m*/*z* 64.01419 ± 5 ppm, *m*/*z* 64.02506 ± 5 ppm and *m*/*z* 68.05614 ± 5 ppm, respectively.

### Quantitative reverse transcription polymerase chain reaction assays

*Halomonas* sp. HTNK1 was grown at 30 °C in MBM 35 PSU and 10 mM succinate (control) or succinate plus 5 mM DMSP, DMSOP or acrylic acid (Fisher Scientific; 164252500) in triplicate until mid-exponential phase (OD_600_ of 0.4). Total RNA was extracted using a RNeasy mini kit (QIAgen; 74106) and reverse transcription was performed with the QuantiTect reverse transcription kit (QIAgen; 205313) following the manufacturer’s instructions. Primers used in quantitative reverse transcription polymerase chain reactions (RT–qPCRs) are listed in Supplementary Table 7. RT–qPCR assays were performed on an AriaMx Real-Time PCR system (Agilent) with the PerfeCTa qPCR SuperMix (Quantabio; 95054-02K) and the following cycling conditions: 95 °C for 3 min, 40 cycles of 95 °C for 20 s, 60 °C for 30 s and 72 °C for 30 s.

*P. ubique* HTCC1062 was grown in artificial seawater with pyruvate and methionine as carbon and sulfur source, respectively. When cultures reached 8 × 10^6^ to 1 × 10^7^ cells ml^−^^1^, cells were induced with 100 µM DMSP or DMSOP. Cultures with no DMSP or DMSOP added were set up as controls. Each condition was set up in triplicate. Total RNA was extracted using a RNeasy mini kit (QIAgen; 74106) following the manufacturer’s instructions and reverse transcribed with a PrimeScrip RT Reagent Kit with gDNA Eraser (TaKaRa; RR047A). RT–qPCRs were performed on a Light Cycler II 480 System (Roche) with SYBR Premix Ex TaqTM (TaKaRa; DRR420A) and the following cycling conditions: 95 °C for 5 min, 45 cycles of 95 °C for 10 s and 60 °C for 30 s.

### Gene cloning, point mutation and protein expression and purification

The *ddd* and *Alma* genes listed in Supplementary Table 8 were cloned into Novagen pET-22b vector (Merck; 69744) with a C-terminal His tag. Site-directed mutagenesis was performed using PCR-based methods with the Quick-change mutagenesis kit II (Agilent; 200518), and mutants were verified by DNA sequencing. WT and mutant proteins were expressed in *E. coli* BL21(DE3). Cells were grown in LB medium^47^ at 37 °C to an OD_600_ of 0.8–1.0 and then induced at 18 °C for 14–16 h with 0.5 mM isopropyl β-d-1-thiogalactopyranoside. Cells were spun down, resuspended in buffer (50 mM of Tris–HCl, 100 mM of NaCl, 0.5% of glycerol, pH 8.0) and disrupted using a pressure crusher (JNBIO). Proteins were purified at 4 °C by affinity chromatography with Ni^2+^–nitrilotriacetic acid resin (QIAgen) using 50 mM Tris-HCl, 100 mM NaCl and 20 mM imidazole (pH 8.0) as a wash buffer and 50 mM Tris–HCl, 100 mM NaCl and 250 mM imidazole (pH 8.0) as an elution buffer. Purified proteins were further fractionated by gel filtration on Superdex-75 and 200 columns (GE Healthcare) using 10 mM Tris–HCl and 100 mM NaCl (pH 8.0) as an elution buffer.

### Enzymatic activity assays

To test DMSOP lyase activities, 0.15–1.5 µM of purified enzymes were mixed with the reaction buffer containing 100 mM Tris–HCl (pH 8.0) and 5 mM DMSOP. For DddX, the reaction buffer was composed of 1 mM CoA, 1 mM adenosine triphosphate, 2 mM MgCl_2_, 50 mM Tris–HCl (pH 8.0) and 5 mM DMSOP. After incubation at 30 °C, the reaction was stopped by adding 10% (v/v) perchloric acid. Reaction buffers with no enzymes were used as negative controls. DMSO in reaction mixtures was detected by high-performance liquid chromatography (HPLC) using an Ultimate 3000, Dionex and LC-20AT instrument (Shimadzu) with a SunFire C18 column (Waters) and a constant flow of 100 mM ammonium dihydrogen phosphate (pH 2.5) over 20 min at 210 nm. DMSO generated by DMSP lyases was quantified using standards ranging from 0 to 1 mM. Acrylate production from DMSOP was detected by measuring its ultraviolet absorbance at 210 nm by HPLC and quantified using standards ranging from 0 to 10 mM.

DMSP lyase activity of Ddd enzymes was examined as above using 5 mM of DMSP instead of DMSOP and measuring the ultraviolet absorbance of acrylate by HPLC.

To identify the DddX DMSP and DMSOP cleavage products, the reaction mixtures were simultaneously analysed by LC mass spectrometry as in Li et al.^26^.

For in vitro DMSP and DMSOP lyase competition experiments, 10 mM of DMSP and DMSOP were added simultaneously to the reaction systems containing the purified proteins. Resultant DMSO, acrylate and ADP (for DddX catalysis) were detected by HPLC as above. The DMSO levels detected in the reactions reported the DMSOP lyase activity, which when subtracted from the detected levels of acrylate (from both DMSOP and DMSP cleavage) allowed the calculation of DMSP cleavage levels. With the tested enzymes, DMSP cleavage was always higher than DMSOP cleavage activity, and the latter was presented as a percentage of total DMSP lyase activity (as relative activities).

To determine the optimal temperature of DddY for DMSOP, reaction mixtures were incubated at 0–70 °C for 10 min. Optimum pH of DddY for DMSOP was examined at its optimal temperature using Bis–Tris buffer for pH 5–7, Tris buffer for pH 7–9 and glycine buffer for pH 9–10.

Kinetic parameters of DMSP lyases listed in Supplementary Table 1 for DMSOP were determined by non-linear analysis based on the initial rates of acrylate production (DMSO production for DddX) at 30 °C and pH 8.

Enzymatic activity assays results represent the mean of triplicate experiments with their respective s.d.

### Crystallization and data collection

Before crystallization, the purified DddK mutant Tyr64Ala and DddY mutant Tyr260Ala were concentrated to ~20 mg ml^−^^1^ in 10 mM Tris–HCl buffer (pH 8.0) with 100 mM NaCl. To obtain the structure of the DddK/DMSOP and DddY/DMSOP complexes, mutants were co-crystallized with 1 mM DMSOP. Crystallization trials for DddK–DMSOP and DddY–DMSOP complexes were performed at 18 °C using the sitting-drop vapour diffusion method. Diffraction-quantity crystals of the DddK–DMSOP complex were obtained in sitting drops containing 0.1 M succinic acid, sodium dihydrogen phosphate and glycine buffer (pH 5.0) and 25% (w/v) polyethylene glycol 1500. The buffer was produced by mixing succinic acid, sodium dihydrogen phosphate and glycine in a 2:7:7 molar ratio. Diffraction-quantity crystals of DddY–DMSOP complex were obtained in sitting drops containing 0.1 M Tris–HCl (pH 8.5) and 25% polyethylene glycol 3350. Crystals were collected after a 2-week incubation at 18 °C. X-ray diffraction data were collected on the BL18U1 beamline at the Shanghai Synchrotron Radiation Facility. The initial diffraction data were processed using the HKL3000 software v715 with default settings^60^.

### Structure determination and refinement

All crystals of DddK–DMSOP and DddY–DMSOP complexes belonged to the P21 space group. The crystal structures of DddK–DMSOP and DddY–DMSOP complexes were determined by molecular replacement using the CCP4 phaser program v6.5 (ref. ^61^). Structure refinement was performed with WinCoot v0.8.1 (ref. ^62^) and Phenix v1.16-3549 (ref. ^63^). All software was used with default parameters and structure figures processed with PyMOL v1.6.0.0 (http://www.pymol.org/).

### Circular dichroism spectroscopy

Circular dichroism spectroscopic assays of WT and mutant DddK proteins were performed at 25 °C on a J-1500 CD spectrometer (Jasco). All spectra were collected from 250 to 200 nm at a scan speed of 500 nm min^−^^1^ with a band width of 1 nm using 10 μM protein in distilled water.

### Molecular docking

The PDB format of DMSOP’s chemical structure was acquired using Chem Draw and Chem 3D v17.1.0.105 (Perkin Elmer). The crystal structures of DddP–acrylate (PDB code: 4S01) and DddQ–DMSP (PDB code: 4LA3) were obtained from RCSB PDB database (http://www.rcsb.org/). Molecular docking was performed using the docking tool in the Zcloud platform (https://cloud.zelixir.com).

### Relative abundance and expression of DMSP lyases genes in Tara Oceans datasets

The distribution of *ddd*, *Alma* and *dmdA* genes in the global ocean was estimated by analysing the Tara Oceans datasets OM-RGC-v2 (ref. ^64^) and MATOU^65^. This analysis was conducted using the online webserver Ocean Gene Atlas v2.0 (ref. ^66^) using hmmsearch with an e-value of <1 × 10^−30^.

Briefly, hmm databases based on ratified sequences of these genes^67^ were submitted to Ocean Gene Atlas to detect homologues in OM-RGC-v2 and MATOU metagenomes/metatranscriptomes. Resultant sequences were subjected to a further BLASTp analysis. Only homologues with ≥40% amino acid identity and ≥70% coverage to ratified sequences were counted. In metagenomic samples, relative abundances of *Alma* genes were normalized to the relative abundance of *ACTB* genes (a phylogenetic marker gene for eukaryotes, encoding β-actin protein), whereas relative abundances of *ddd* and *dmdA* genes were normalized to the average relative abundance of ten conserved single-copy marker genes as in Liu et al.^67^. *ACTB* and ten marker genes were retrieved using the method detailed above and a hmmsearch e-value of <1 × 10^−10^. In metatranscriptomic samples, the relative abundance of *ddd*, *dmdA* and *Alma* transcripts is expressed as percentage of mapped reads. In MATOU datasets, only the 0.8–20 µm fraction (picoplankton/nanoplankton) was analysed.

### Reporting summary

Further information on research design is available in the Nature Portfolio Reporting Summary linked to this article.

### Supplementary information

Supplementary Information Supplementary Figs. 1–14 and Tables 1–8.

Reporting Summary

### Source data

Source Data Fig. 1 Validation report of DddK–DMSOP complex structure.

Source Data Fig. 2 Validation report of DddY–DMSOP complex structure.

## Data Availability

The structures of DddK–DMSOP and DddY–DMSOP complexes generated in this study are publicly available from the Protein Data Bank (PDB) under accession numbers 8HLF and 8HLE. Validation reports of DddK–DMSOP and DddY–DMSOP complex structures are provided as Source Data Figs. 1 and 2, respectively. Source data are provided with this paper.
