# Supplementary material for: DMSOP-cleaving enzymes are diverse and widely distributed in marine microorganisms
Source: Nat Microbiol. 2023 Nov 29;8(12):2326–37. doi: 10.1038/s41564-023-01526-4 (PMC10686828; doi:10.1038/s41564-023-01526-4)
Supplement: Supplementary file 1 — Supplementary Figs. 1–14 and Tables 1–8. [file 41564_2023_1526_MOESM1_ESM.pdf]

nature microbiology

Article

https://doi.org/10.1038/s41564-023-01526-4

DMSOP-cleaving enzymes are diverse and widely distributed in marine microorganisms

In the format provided by the authors and unedited

Supplementary information

DMSOP-cleaving enzymes are diverse and widely distributed in marine microorganisms

Ornella Carrión[1,2]†*, Chun-Yang Li[1]†*, Ming Peng[1,3]†, Jinyan Wang[1], Georg Pohnert[4], Muhaiminatul Azizah[4], Xiao-Yu Zhu[2], Andrew R.J. Curson[2], Qing Wang[3], Keanu S. Walsham[2], Xiao-Hua Zhang[1], Serena Monaco[5], James M. Harvey[6], Xiu-Lan Chen[3,7], Chao Gao[3], Ning Wang[3], Xiu-Juan Wang[3], Peng Wang[1], Stephen J. Giovanonni[8], Chih-Ping Lee[8], Christopher P. Suffridge[8], Yu Zhang[9], Ziqi Luo[9], Dazhi Wang[9], Jonathan D. Todd[1,2]* and Yu-Zhong Zhang[1,3,10,11]*

1 MOE Key Laboratory of Evolution and Marine Biodiversity, Frontiers Science Center for Deep Ocean Multispheres and Earth System & College of Marine Life Sciences, Ocean University of China, Qingdao 266003, China.

2 School of Biological Sciences, University of East Anglia, Norwich Research Park, Norwich NR4 7TJ, UK.

3 State Key Laboratory of Microbial Technology, Marine Biotechnology Research Center, Shandong University, Qingdao, 266237, China.

4 Institute of Inorganic and Analytical Chemistry, Bioorganic Analytics, Lessingstr. 8, Friedrich Schiller University Jena, 07743 Jena, Germany.

5 School of Pharmacy, University of East Anglia, Norwich Research Park, Norwich NR4 7TJ, UK.

6 Department of Chemistry, King's College London, London, UK.

7 Laboratory for Marine Biology and Biotechnology, Pilot National Laboratory for Marine Science and Technology (Qingdao), Qingdao 266373, China.

8 Department of Microbiology, Oregon State University, Corvallis, 97331, Oregon, USA.

9 State Key Laboratory of Marine Environmental Science/College of the Environment & Ecology, Xiamen University, Xiamen 361102, China.

10 Frontiers Science Center for Deep Ocean Multispheres and Earth System, Qingdao 266373, China.

11 Joint Research Center for Marine Microbial Science and Technology, Shandong University and Ocean University of China, Qingdao, 266237, China.

*Corresponding authors: Yu-Zhong Zhang; Chun-Yang Li; Ornella Carrión; Jonathan D. Todd.

Email: zhangyz@sdu.edu.cn; Lcy@ouc.edu.cn; O.Carrion-Fonseca@uea.ac.uk; jonathan.todd@uea.ac.uk

Classification: Biological sciences; Environmental sciences

Keywords: Dimethylsulfoxonium propionate; dimethylsulfoniopropionate; dimethyl sulfoxide; DMSP lyases; marine sulfur cycle

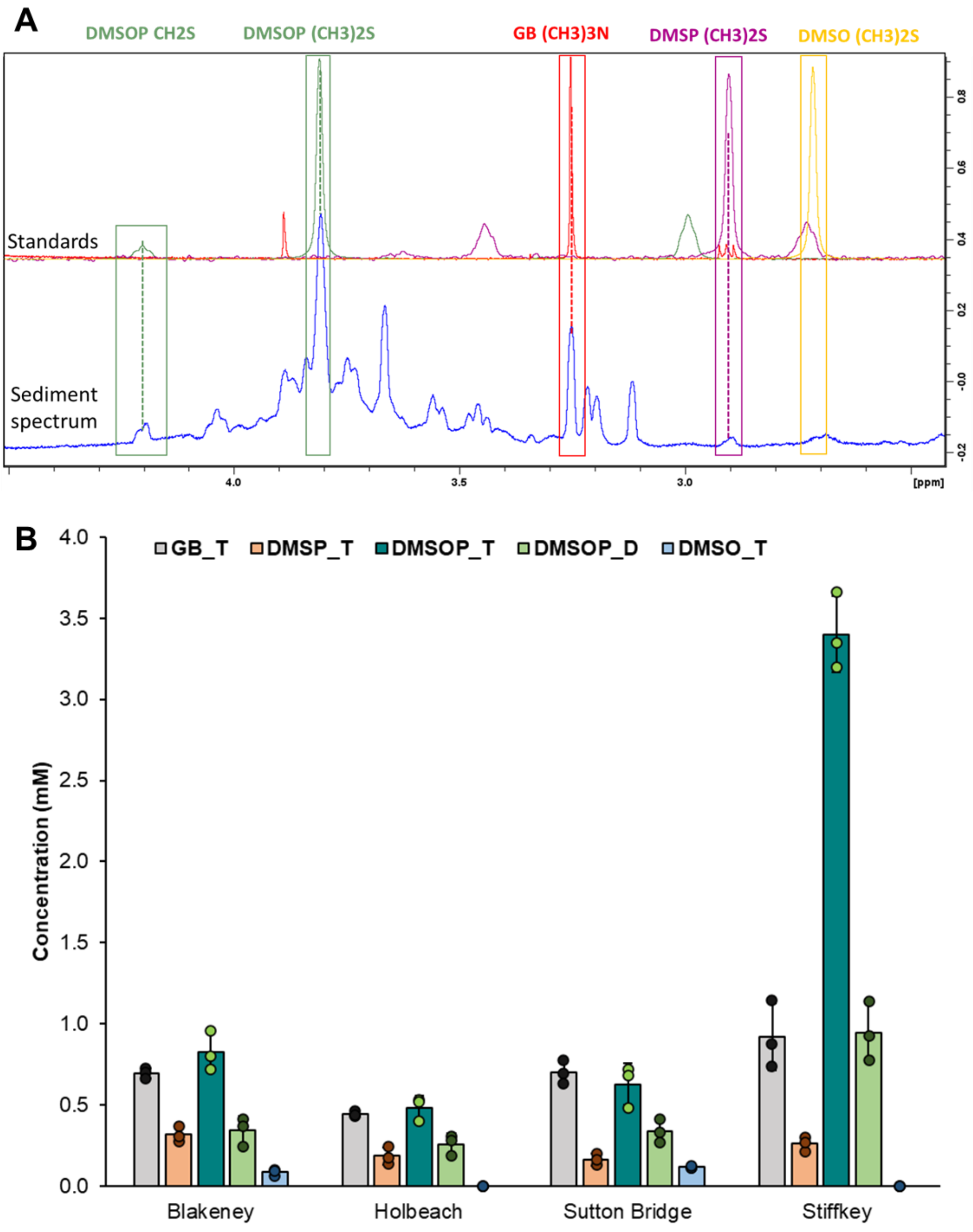

Fig S1. Quantification of DMSP, DMSOP, DMSO and GB in saltmarsh sediments by NMR. A; NMR spectra of DMSP, DMSOP, DMSO and GB standards and Stiffkey saltmarsh sediment. B; Concentrations of key organosulfur compounds and GB in marine sediments using pyrazine as internal standard (see Methods). T: total; D: dissolved. Data are presented as mean ± sd (n=3).

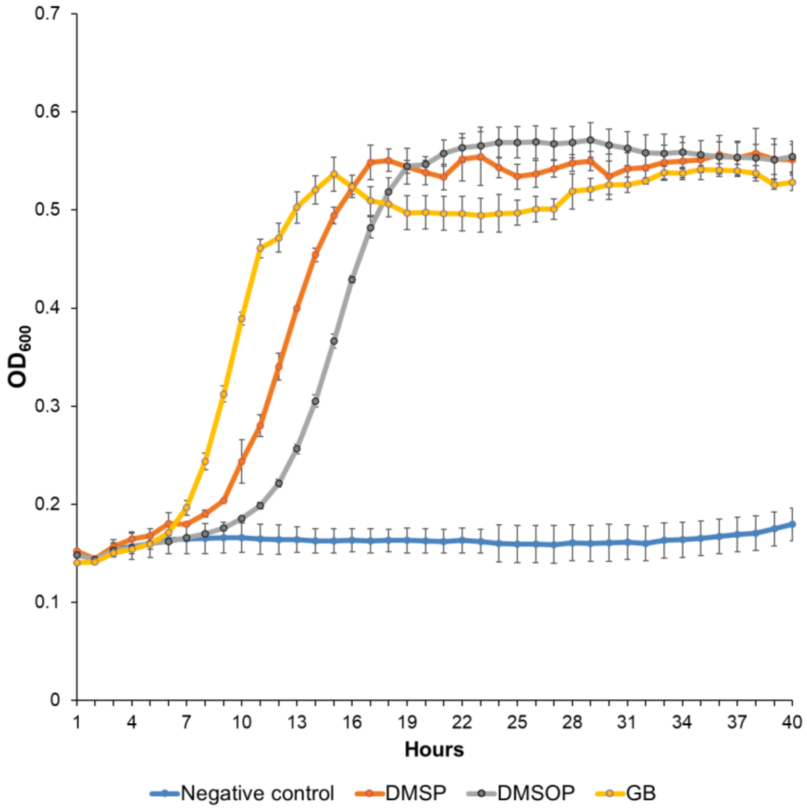

Fig S2. DMSP and DMSOP confer osmotolerance to *E. coli* FF4169. *E. coli* FF4169, deficient in trehalose production, was grown in M63 medium containing 0.5 M NaCl plus 1 mM GB, DMSP, DMSOP or no additives as negative control. Data are presented as mean ± sd (n=3).

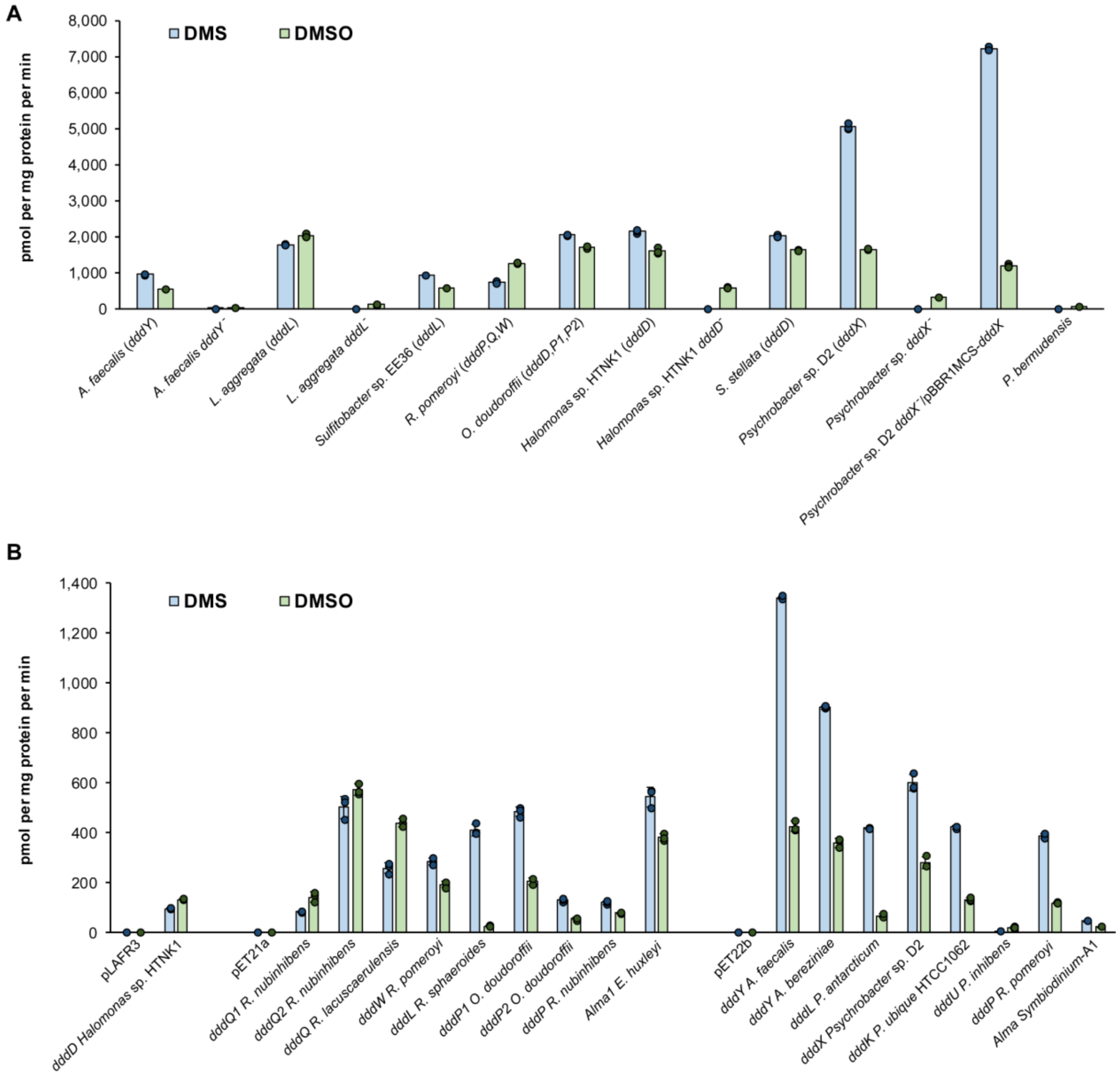

Fig S3. In vivo DMSP and DMSOP lyase activity of bacterial strains. A; DMSP and DMSOP lyase activities of marine strains containing functional DMSP lyases and their respective *ddd*$^{-}$ mutant strains with either DMSP or DMSOP added (to 0.5 mM). *P. bermudensis*, which has no DMSP lyase activity and no DMSP lyase enzymes, was used as negative control. The complement of *ddd* genes found in tested bacteria are indicated in brackets. B; DMSP and DMSOP lyase activities of Ddd and Alma enzymes expressed in *E. coli* BL21 with either 0.5 mM DMSP or DMSOP added. *E. coli* BL21 containing empty vectors were used as negative controls. Data are presented as mean ± sd (n=3).

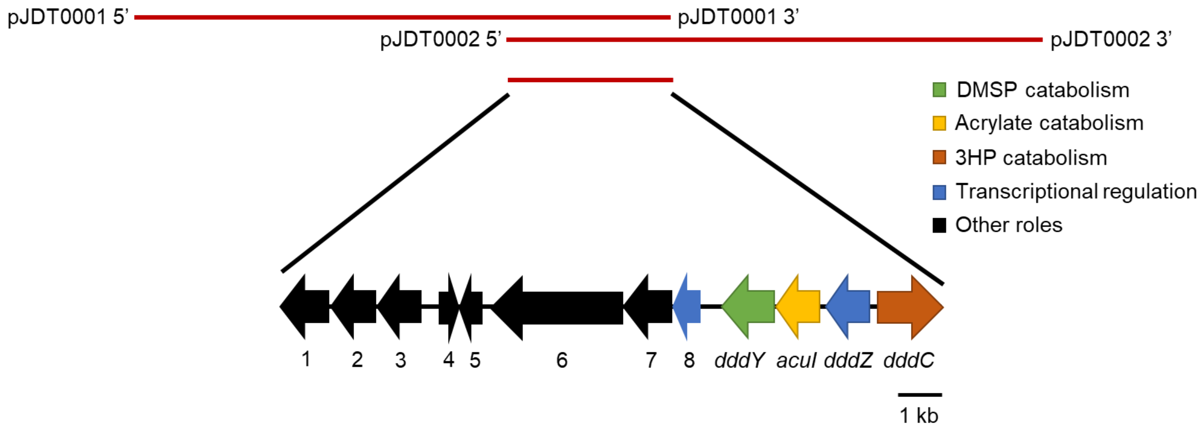

Fig S4. The 15 kb overlapping *A. faecalis* M3A genomic region cloned in pJDT0001 and pJDT0002 that conferred DMSOP lyase activity. Predicted gene products: 1. Purine-binding protein; 2. EamA/RhaT family transporter; 3. Biotin synthase; 4. 4-hydroxybenzoyl-CoA thioesterase; 5. DUF3293 domain containing-protein; 6. Multidrug/solvent efflux pump membrane transporter MepB; 7. Multidrug resistance protein MdtE; 8. TetR/AcrT family transcriptional regulator; *dddY*. DMSP lyase DddY; *acuI.* Acrylyl-coA reductase; *dddZ*. LysR transcriptional regulator; *dddC*. Aldehyde dehydrogenase. 3HP; 3-hydroxypropionate.

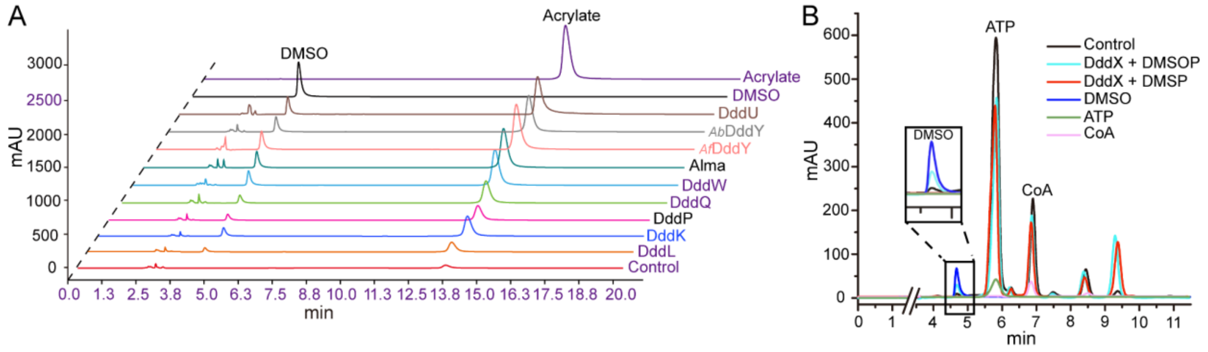

Fig. S5. In vitro DMSOP lyase activity of Ddd and Alma enzymes detected by HPLC. A; In vitro production of DMSO and acrylate by purified cupin DMSP lyases, DddP and Alma. B; *In vitro* DMSOP lyase activity of the purified *Psychrobacter* sp. D2 DddX. Reaction mixtures with no enzymes were used as negative controls. $_{Ab}$DddY: DddY from *A. bereziniae*; $_{Af}$DddY: *A. faecalis* DddY.

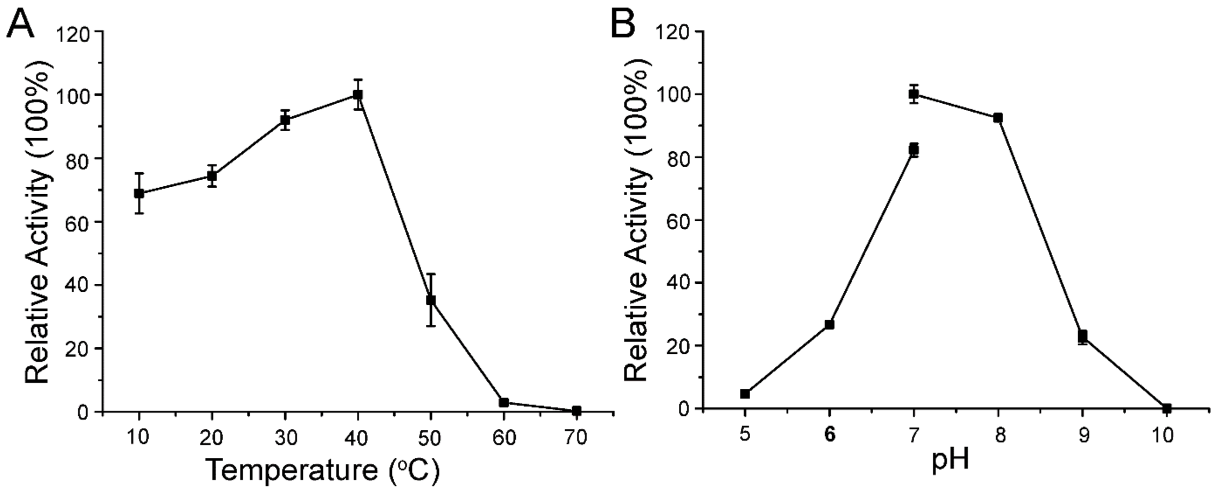

Fig. S6. Characterization of purified *A. faecalis* DddY. A; Effect of temperature on DddY DMSOP cleavage activity. DMSOP cleavage activity at 40 ºC was defined as 100%. B; Effect of pH on DddY DMSOP cleavage activity. DMSOP cleavage activity at pH 7.0 was defined as 100%. Data are presented as mean ± sd (n=3).

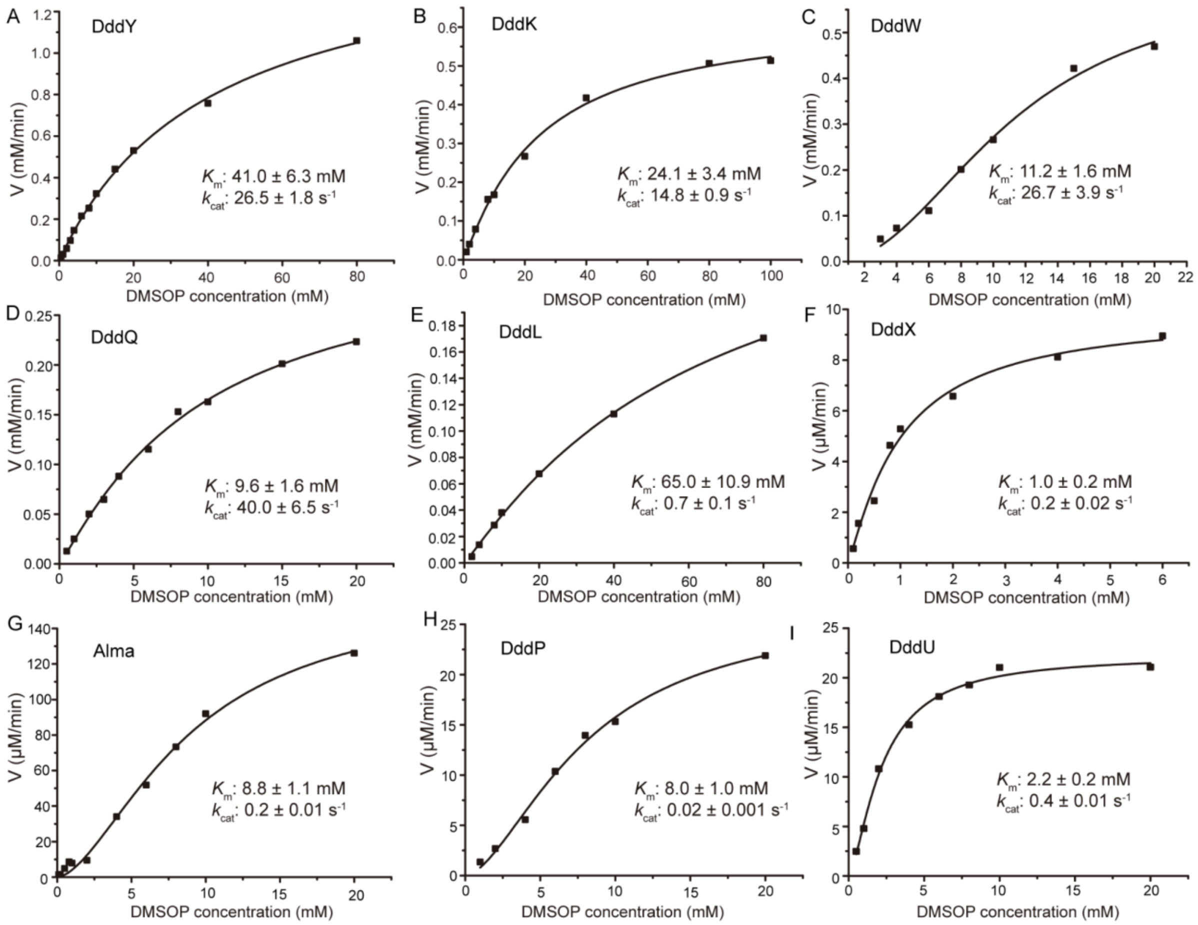

Fig S7. Enzyme kinetics of purified Ddd and Alma enzymes for DMSOP substrate. A; *A. faecalis* DddY. B; *P. ubique* HTCC1062 DddK. C; *R. pomeroyi* DddW. D; *R. lacuscaerulensis* DddQ. E; *P. antarcticum* DddL. F; *Psychrobacter* sp. D2 DddX. G; *Symbiodinium*-A1 Alma (Clade A). H; *O. doudoroffii* DddP1. I; *P. inhibens* DddU. Kinetic parameters of tested DMSP lyase enzymes were determined by non-linear analysis based on the initial rates of acrylate or DMSO (DddX) production in triplicate experiments.

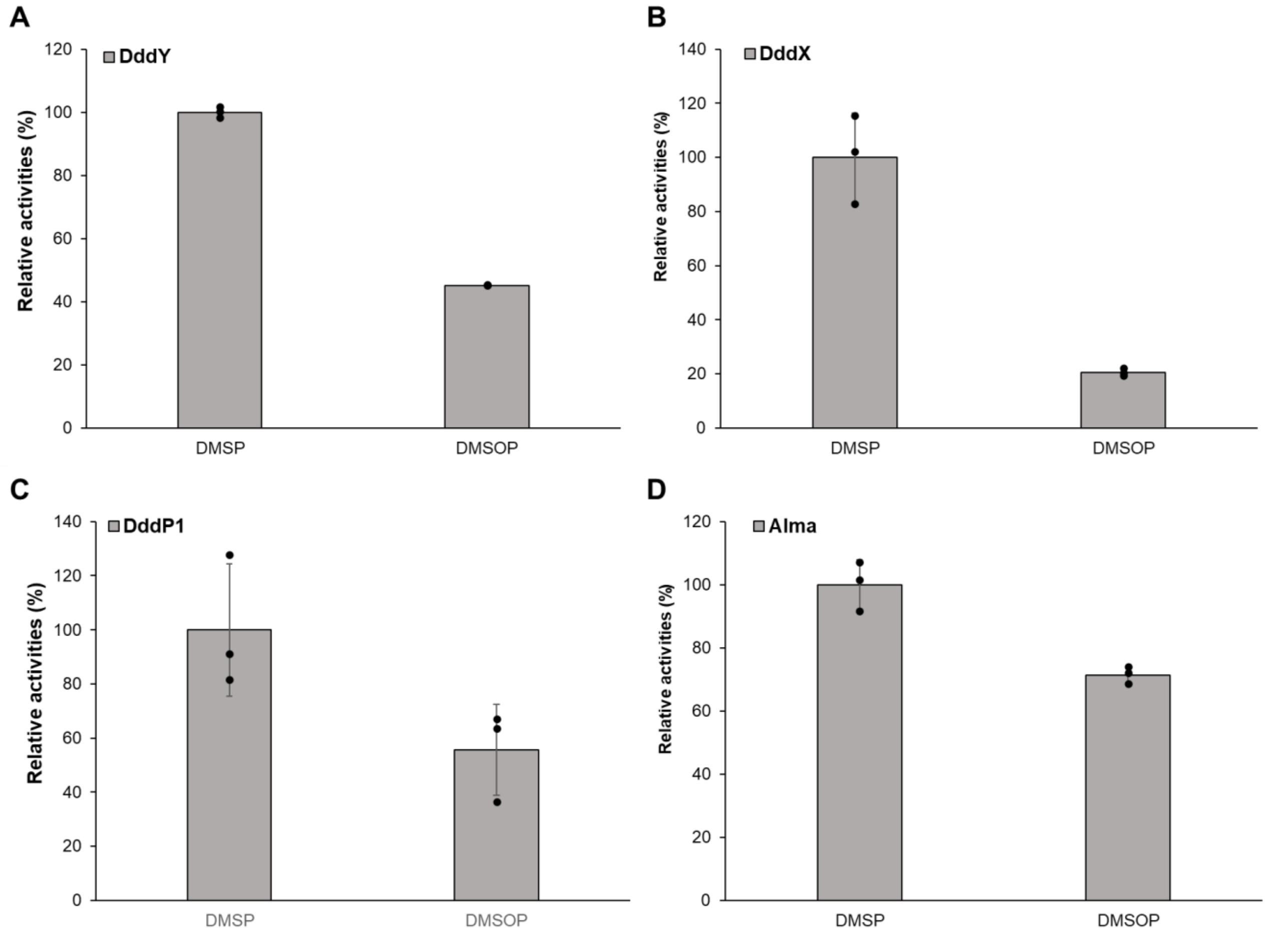

Fig. S8. In vitro DMSP and DMSOP lyase activity assays with purified DMSP lyase enzymes and equimolar levels of DMSP and DMSOP. Pure DMSP lyase protein was incubated with both DMSP and DMSOP each at 10 mM concentration. DMSOP lyase activity is expressed as a percentage of the DMSP lyase activity that was always superior with the enzymes tested here. A; *A. faecalis* DddY. B; *Psychrobacter* sp. D2 DddX. C; *O. doudoroffii* DddP1. D; *Symbiodinium*-A1 Alma (Clade A). Data are presented as mean ± sd (n=3).

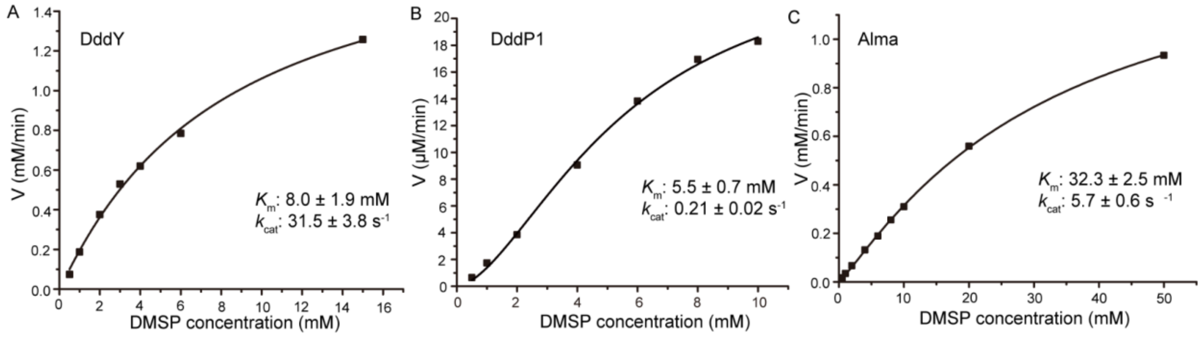

Fig. S9. Enzyme kinetics of purified Ddd and Alma enzymes for DMSP substrate. A; *A. faecalis* DddY. B; *O. doudoroffii* DddP1. C; *Symbiodinium*-A1 Alma (Clade A). Kinetic parameters of tested Ddd enzymes were determined by non-linear analysis based on the initial rates of acrylate production in triplicate experiments.

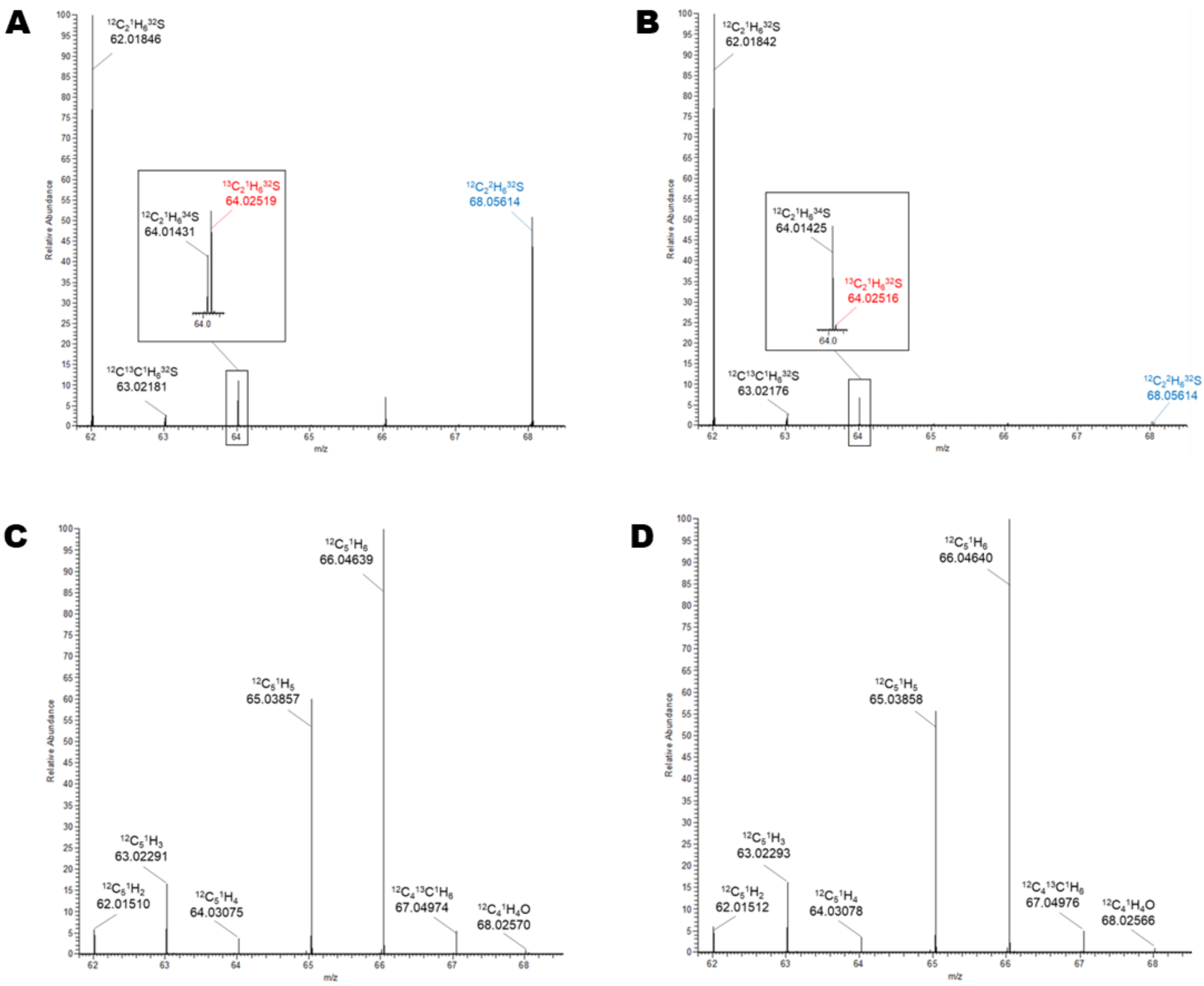

Fig S10. DMSOP and DMSP lyase activity of *Emiliana huxleyi* analysed by GC-HRMS. A; High-resolution mass spectrum of DMS obtained from *E. huxleyi* RCC173/CCMP373 incubated with $^{13}C_2$-labelled DMSOP, $^2H_6$-labelled DMSP and $TiCl_3$ added as reducing agent. B; High-resolution mass spectrum of DMS obtained from *E. huxleyi* RCC173/CCMP373 incubated with $^{13}C_2$-labelled DMSOP, $^2H_6$-labelled DMSP, Br-DMSP 50µM and $TiCl_3$. C; Abiotic control with $^{13}C_2$-labelled DMSOP, $^2H_6$-labelled DMSP and $TiCl_3$. D; Abiotic control with $^{13}C_2$-labelled DMSOP, $^2H_6$-labelled DMSP, Br-DMSP 50µM and $TiCl_3$. Peaks of $^{13}C_2$-labelled DMS generated from DMSOP and $^2H_6$-labelled DMS produced from DMSP are represented in red and blue, respectively. The natural DMS isotopes are shown in black. All experiments were conducted in triplicate.

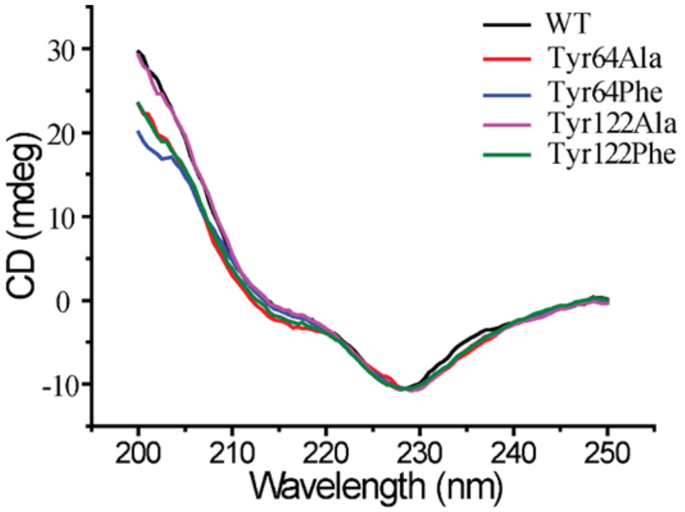

Fig. S11. Circular-dichroism (CD) spectra of wild type (WT) and mutant DddK proteins. Spectra of all proteins were collected from 250 to 200 nm at a scan speed of 500 nm·min^{-1} with a band width of 1 nm to determine their secondary structure. The specific amino acid substitution mutations are indicated.

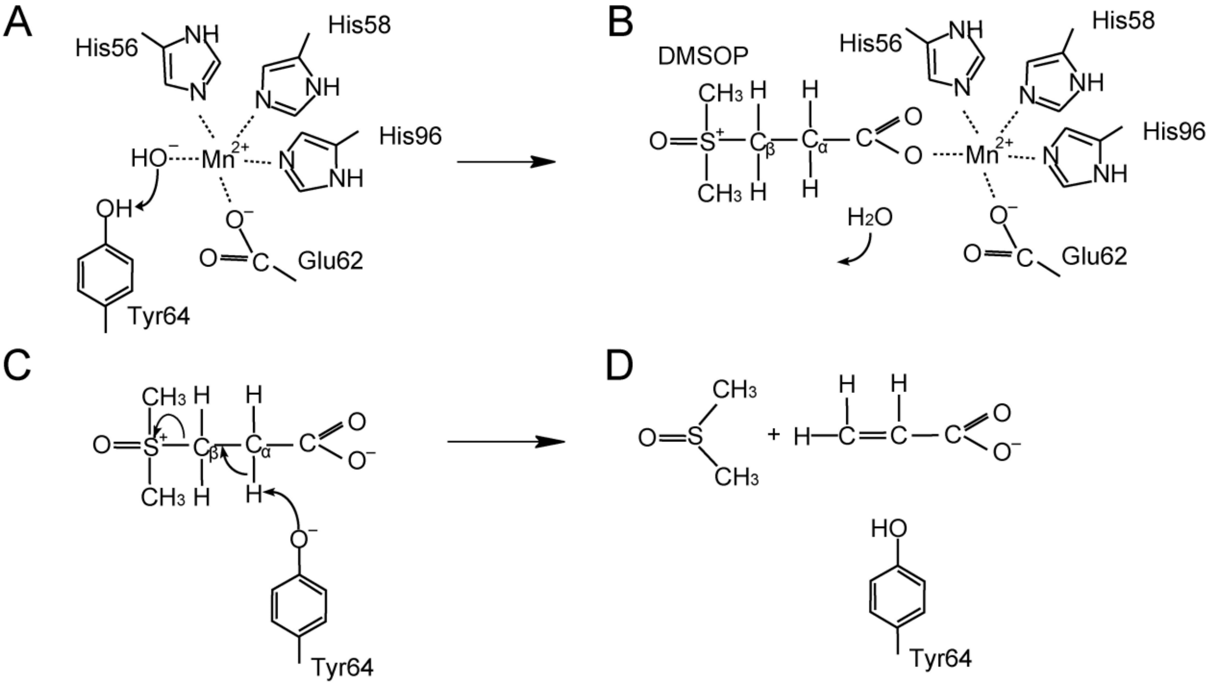

Fig. S12. The proposed DddK catalytic mechanism of DMSOP cleavage. A; In the absence of DMSOP, Mn^{2+} is coordinated by residues His56, His58, Glu62, His96 and a water molecule. The residue Tyr64 forms a hydrogen bond with the water molecule activated by Mn^{2+}, which may help the deprotonation of Tyr64 to act as a catalytic base. B; DMSOP replaces the water molecule and forms a new coordination bond with Mn^{2+}. C; The Tyr64 residue acts as a general base to attack DMSOP. D; DMSO and acrylate are generated from DMSOP cleavage.

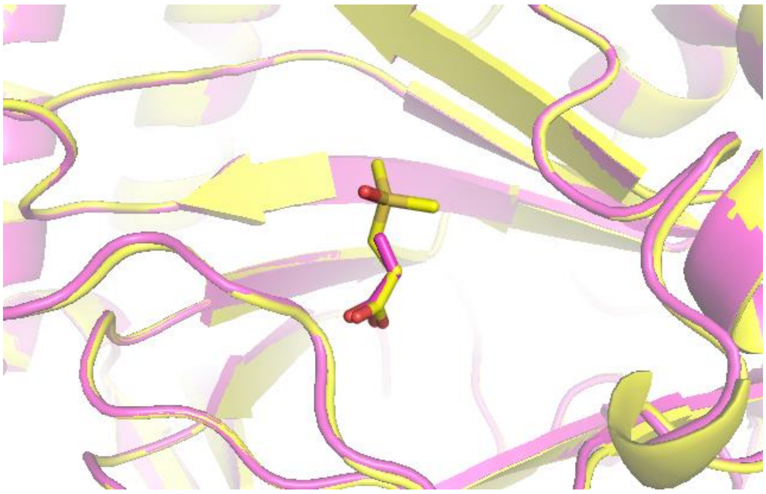

Fig. S13. Structural alignment of DddY from *A. bereziniae* (*Ab*DddY) with DMSOP and acrylate (PDB code: 5Y4K). The structure of the *Ab*DddY-DMSOP complex is coloured in yellow, whereas the structure of the *Ab*DddY-acrylate complex is shown in magenta. The DMSOP and acrylate molecules are shown as sticks.

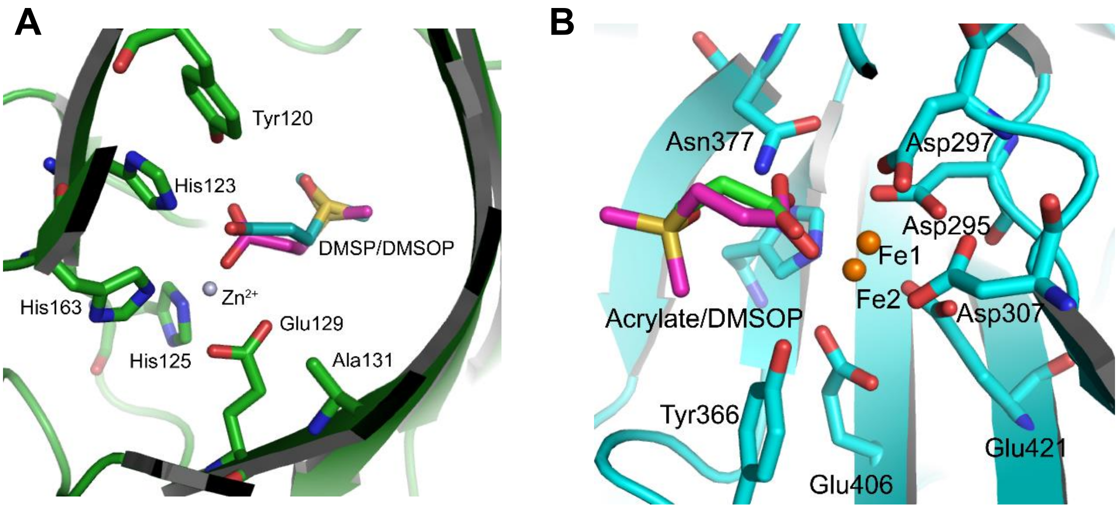

Fig. S14. Structural analyses of DddQ-DMSOP and DddP-DMSOP complexes. The structures of DddQ-DMSOP and DddP-DMSOP complexes were obtained by molecular docking. A; Structural alignment of DddQ-DMSOP and DddQ-DMSP complex (PDB: 4LA3). The structure of DddQ is coloured in green. DMSOP is shown as magenta sticks, and DMSP as cyan sticks. B; Structural alignment of DddP-DMSOP and DddP-acrylate complex (PDB code: 4S01). The structure of DddP is coloured in cyan. DMSOP is shown as magenta sticks, and acrylate as green sticks.

Table S1. Kinetic parameters of DMSP lyases with DMSOP and DMSP substrates.

Enzyme	Origin	DMSOP			DMSP		
		K_m (mM)*	k_{cat} (s^{-1})*	k_{cat}/K_m ($mM^{-1}s^{-1}$)	K_m (mM)*	k_{cat} (s^{-1})*	k_{cat}/K_m ($mM^{-1}s^{-1}$)
DddY	*Alcaligenes faecalis*	41.0 ± 6.3	26.5 ± 1.8	0.64	8.0 ± 1.9	31.5 ± 3.8	3.94
DddK	*Pelagibacter ubique* strain HTCC1062	24.1 ± 3.4	14.8 ± 0.9	0.61	13.6 ± 2.1[1]	2.1 ± 0.1[1]	0.15
DddW	*Ruegeria pomeroyi* DSS-3	11.2 ± 1.6	26.7 ± 3.9	2.38	8.7 ± 0.7[2]	18.3[2]	2.10
DddQ	*Ruegeria lacuscaerulensis* ITI_1157	9.6 ± 1.6	40.0 ± 6.5	4.17	21.5 ± 6.8[3]	1.0 ± 0.3[3]	0.05
DddL	*Puniceibacterium antarcticum* SM1211	65.0 ± 10.9	0.7 ± 0.1	0.01	2.82[4]	0.09[4]	0.03
DddX	*Psychrobacter* sp. D2	1.0 ± 0.2	0.2 ± 0.02	0.20	0.4 ± 0.03[5]	0.7 ± 0.02[5]	1.75
Alma (Clade A)	*Symbiodinium*-A1	8.8 ± 1.1	0.2 ± 0.01	0.02	32.3 ± 2.5	5.7 ± 0.6	0.18
DddP1	*Oceanimonas doudoroffii*	8.0 ± 1.0	0.02 ± 0.001	0.003	5.5 ± 0.7	0.2 ± 0.02	0.04
DddU	*Phaeobacter inhibens* P66	2.2 ± 0.2	0.4 ± 0.01	0.18	8.0 ± 0.8[6]	0.7 ± 0.02[6]	0.09

* Mean of three biological replicates (n=3) with standard deviations shown.

Table S2. Crystallographic data collection and refinement of DddK from *P. ubique* HTCC1062 and DddY from *A. bereziniae* with DMSOP.

Parameters	DddK-DMSOP complex	DddY-DMSOP complex
**Diffraction data**		
Space group	$P2_1$	$P2_1$
Unit cell		
a, b, c (Å)	36.8, 92.8, 38.8	65.8, 73.4, 87.4
α, β, γ (°)	90.0, 117.8, 90.0	90.0, 91.1, 90.0
Resolution range (Å)	50.0-1.6 (1.68-1.62) *	50.0-1.9 (1.97-1.90) *
Redundancy	3.4 (3.4)	6.6 (6.7)
Completeness (%)	98.5 (97.2)	98.1 (97.1)
R_{merge}**	0.072 (0.19)	0.20 (0.58)
$I/\sigma I$	22.5 (6.4)	23.3 (7.7)
**Refinement statistics**		
R-factor	0.18	0.16
Free R-factor	0.20	0.20
RMSD from ideal geometry		
Bond lengths (Å)	0.015	0.007
Bond angles (°)	1.44	0.9
Ramachandran plot (%)		
Favored	100	97.6
Allowed	0	2.3
Outliers	0	0.1
Overall B-factors (Å^2)	17.9	19.2

*Numbers in parentheses refer to data in the highest-resolution shell.

**$R_{merge}=\sum_{hkl}\sum_i|I(hkl)_i - \langle I(hkl)\rangle|/\sum_{hkl}\sum_i I(hkl)_i$, where I is the observed intensity, $\langle I(hkl)\rangle$ represents the average intensity, and $I(hkl)_i$ represents the observed intensity of each unique reflection.

Table S3. Relative abundances of DMSP/DMSOP lyase and *dmdA* genes and transcripts in Tara Ocean datasets. In metagenomic data, relative abundance of prokaryotic and eukaryotic genes were normalised to *ACTB* and 10 prokaryotic single copy genes, respectively (see Methods). Relative abundance of transcripts in metatranscriptomics data is expressed as percentage of mapped reads. SRF, surface water layer; DCM, deep chlorophyll maximum layer; MES, mesopelagic water layer.

Database	Sequencing type	Layer	prokaryotic DMSP lyase genes										prokaryotic DMSP demethylation genes
			dddD	*dddK*	*dddL*	*dddP*	*dddQ*	*dddU*	*dddW*	*dddX*	*dddY*	**Total**	*dmdA*
OM-RGC_v2	metagenome (percent of prokaryotic cells)	SRF	2.3E-01	1.7E+00	1.6E-02	5.3E+00	1.5E+00	4.4E-01	5.2E-03	8.7E-01	0	1.0E+01	3.0E+01
		DCM	3.0E-01	1.4E+00	1.9E-02	6.2E+00	1.1E+00	5.0E-01	2.0E-03	8.5E-01	0	1.0E+01	2.9E+01
		MES	1.2E-01	4.4E-01	3.7E-02	1.1E+01	6.9E-01	2.0E-01	3.3E-04	6.0E-01	0	1.3E+01	1.8E+01
	metatranscriptome (percent of mapped reads)	SRF	1.4E-06	1.9E-06	3.9E-08	6.4E-06	1.1E-06	3.6E-07	9.7E-09	2.9E-06	0	1.4E-05	6.8E-05
		DCM	9.5E-07	1.2E-06	1.0E-07	5.9E-06	7.3E-07	3.5E-07	3.7E-09	1.9E-06	0	1.1E-05	5.1E-05
		MES	8.5E-07	2.7E-07	3.6E-07	8.4E-06	3.4E-07	1.4E-07	0	2.3E-07	0	1.1E-05	2.3E-05

			eukaryotic DMSP lyase gene
			Alma
MATOU	metagenome (percent of eukaryotic cells)	SRF	1.2E+00
		DCM	1.3E+00
	metatranscriptome (percent of mapped reads)	SRF	6.3E-06
		DCM	6.9E-06

Table S4. Saltmarsh sediments tested in this study.

Location	Coordinates
Blakeney	52°57'54.5"N; 1°01'01.5"E
Holbeach	52°53'24.6"N; 0°05'31.2"E
Sutton bridge	52°48'59.1"N; 0°13'08.1"E
Stiffkey	52°57'38.4"N; 0°56'11.5"E

Table S5. Strains used in this study.

**Strain**	**Description**	**Reference**
Escherichia coli 803	Strain used for routine transformations	Wood, 1966[7]
E. coli BL21	Strain for overexpression of cloned genes in pET vectors	Studier and Moffat, 1986[8]
E. coli FF4169	Mutant strain deficient in trehalose production that was used in DMSOP osmoprotection work	Giaever *et al*., 1988[9]
Rhizobium leguminosarum J391	Streptomycin-resistant derivative of wild-type strain 3841 used for library screening	Young *et al*., 2006[10]
Alcaligenes faecalis M3A	Wild-type strain with *dddY*	de Souza and Yoch, 1995[11]; Curson *et al*., 2011[12]
Alcaligenes faecalis J482	Rifampicin-resistant derivative of *A. faecalis* M3A with mutation in *dddY*	Curson *et al*., 2011[12]
Labrenzia aggregata LZB033	Wild-type strain containing *dddL*	Curson *et al*., 2017[13]
Labrenzia aggregata J572	Rifampicin-resistant derivative of *L. aggregata* LZB033 with mutation in *dddL*	Curson *et al*., 2017[13]
Sulfitobacter sp. EE36	Wild-type strain with *dddL*	Curson *et al*., 2008[14]
Ruegeria pomeroyi DSS-3	Wild-type strain with *dmdA*, *dddP, dddQ and dddW*	Gonzalez *et al*., 2003[15]; Howard *et al.,* 2006[16]; Todd *et al*., 2012[17]
Oceanimonas doudoroffii DSM 7028	Wild-type strain with dddD, dddP1 and dddP2	Curson *et al*., 2012[18]
Halomonas sp. HTNK1	Wild-type strain with *dddD*	Todd *et al*., 2010[19]
Halomonas sp. J459	Streptomycin-resistant derivative of *Halomonas* sp. HTNK1 with mutation in *dddD*	Todd *et al*., 2010[19]
Sagittula stellata E-37	Wild-type strain containing *dddD*	Gonzalez *et al*., 1997[20]; Johnston *et al*., 2008[21]
Psychrobacter sp. D2	Wild-type strain containing *dddX*	Li *et al*., 2021[5]
Psychrobacter sp. D2 *ΔdddX*	*Psychrobacter* sp. D2 strain with mutation in *dddX*	Li *et al*., 2021[5]
Psychrobacter sp. D2 *ΔdddX/pBBR1MCS-dddX*	*Psychrobacter* sp. D2 *ΔdddX* strain containing cloned *dddX* gene in pBBR1MCS vector	Li *et al*., 2021[5]
Pelagibaca bermudensis HTCC2601	Wild-type strain with no DMSP lyase activity used as negative control	Cho and Giovannoni, 2006[22]
Fusarium culmorum Fu42	Wild-type strain containing *dddP*	Todd *et al*., 2009[23]
Emiliana huxleyi RCC173/CCMP373	Wild-type strain containing *Alma1*	Steinke *et al.,* 1998[24]

Table S6. Plasmids used in this study.

Plasmid	Description	Reference or Accession number
pRK2013	Mobilising plasmid for tri-parental matings (Km^R)	Figurski and Helinski, 1979[25]
pLAFR3	Wide host-range cosmid cloning vector (Tc^R)	Staskawicz *et al*., 1987[26]
pET21a	Plasmid vector for expression of cloned genes in *E. coli* BL21 (Amp^R)	Novagen
pET22b	Plasmid vector for expression of cloned genes in *E. coli* BL21 (Amp^R)	Novagen
pBIO1648	pLAFR3-based cosmid containing 23.7 kb *Halomonas* HTNK1 DNA including *dddD*	Todd et *al*., 2010[19] (FJ849066.1)
pJDT0001	pLAFR3-based cosmid containing 24.7 kp *A. faecalis* M3A DNA including *dddY*	This work
pJDT0002	pLAFR3-based cosmid containing 23.1 kb *A. faecalis* M3A DNA including *dddY*	This work
pJDT0003	pET21a clone containing *dddQ1 from R. nubinhibens* ISM	EAP76002
pJDT0004	pET21a clone containing *dddQ2 from R. nubinhibens* ISM	EAP76001
pJDT0005	pET21a clone containing *dddQ* from *R. lacuscaerulensis* ITI_1157	D0CY60
pJDT0005	pET21a clone containing *dddW* from *R. pomeroyi* DSS-3	AAV93771.1
pJDT0007	pET21a clone containing *dddL* from *R. sphaeroides* 2.4.1	Q3J6L0
pJDT0008	pET21a clone containing *dddP1* from *O. doudoroffii* DSM7028	WP_094198963.1
pJDT0009	pET21a clone containing *dddP2* from *O. doudoroffii* DSM7028	AEQ39103
pJDT0010	pET21a clone containing *dddP* from *R. pomeroyi* DSS-3	WP_044029245
pJDT0011	pET21a clone containing *Alma1* from *E. huxleyi*	XP_005784450
pJDT0012	pET22b clone containing *dddY* from *A. faecalis* M3A	WP_123051132.1
pJDT0013	pET22b clone containing *dddY* from A. bereziniae	WP_004831354.1
pJDT0014	pET22b clone containing *dddL* from *P. antarcticum*	WP_099909581.1
pJDT0015	pET22b clone containing *dddP* from *R.pomeroyi* DSS-3	WP_044029245
pJDT0016	pET22b clone containing *dddX* from *Psychrobacter* sp. D2	PDB: 7CM9
pJDT0017	pET22b clone containing *dddK* from *P. ubique* HTCC1062	WP_011281678.1
pJDT0018	pET22b clone containing *dddU* from *P. inhibens P66*	WP_058277181.1
pJDT0019	pET22b clone containing Clade A *Alma* from *Symbiodinium*-A1	P0DN22.1

*Plasmids pJDT0003-19 were synthesized by Integrated DNA Technologies Ltd (UK) or Beijing Genomics Institute (China) and subcloned into pET21a or pET22b for expression in *E. coli* BL21.

Table S7. Primers used in RT-qPCR assays.

Primer name	Strain	Sequence (5' to 3')
HT_dddD_F	*Halomonas* sp. HTNK1	AGACGCTACGCTCCTACAATGC
HT_dddD_R	*Halomonas* sp. HTNK1	TCCGACACGACGCCATCTTCT
HT_recA_F	*Halomonas* sp. HTNK1	CTCAGGATGACAACCGCACCAA
HT_recA_R	*Halomonas* sp. HTNK1	GCATCGACGAACGCACAGACT
HT_rpoD_F	*Halomonas* sp. HTNK1	ACGATGACGACGAAGACGAGGA
HT_ rpoD_R	*Halomonas* sp. HTNK1	CACGCACCTGCTCAACGCTAA
PU_dddK_F	*P. ubique* HTCC1062	TTATCACTCACCAGCAGAA
PU_dddK_R	*P. ubique* HTCC1062	CAAGGCATGTTCAGCATT
PU_recA_F	*P. ubique* HTCC1062	GCACGAACACAATGATGA
PU_recA_R	*P. ubique* HTCC1062	TGGCACCAATTCTTCTAATG

Table S8. Purified DMSP lyases tested in this study.

Enzyme	Strain	Accession number
Alma (Clade A)	*Symbiodinium*-A1	P0DN22.1
DddK	*Pelagibacter ubique* HTCC1062	WP_011281678.1
DddY	*Acinetobacter bereziniae*	WP_004831354.1
DddY	*Alcaligenes faecalis*	WP_123051132.1
DddQ	*Ruegeria lacuscaerulensis* ITI-1157	D0CY60
DddW	*Ruegeria pomeroyi* DSS-3	AAV93771.1
DddP1	*Oceanimonas doudoroffii*	WP_094198963.1
DddL	*Puniceibacterium antarcticum*	WP_099909581.1
DddX	*Psychrobacter* sp. D2	PDB: 7CM9
DddU	*Phaeobacter inhibens P66*	WP_058277181.1

References

1. Peng, M. *et al.* Structure-Function analysis indicates that an active-site water molecule participates in dimethylsulfoniopropionate cleavage by DddK. *Appl. Environ. Microbiol.* **85,** e03127-18 (2019).

2. Brummett, A. E., Schnicker, N. J., Crider, A., Todd, J. D. & Dey, M. Biochemical, kinetic, and spectroscopic characterization of *Ruegeria pomeroyi* DddW—A mononuclear iron-dependent DMSP lyase. *PLoS One* **10,** e0127288 (2015).

3. Li, C.-Y. *et al.* Molecular insight into bacterial cleavage of oceanic dimethylsulfoniopropionate into dimethyl sulfide. *PNAS.* **111,** 1026-1031 (2014).

4. Teng, Z. J. *et al.* Acrylate protects a marine bacterium from grazing by a ciliate predator. *Nat. Microbiol.* **6,** 1351-1356 (2021).

5. Li, C.-Y. *et al.* A novel ATP dependent dimethylsulfoniopropionate lyase in bacteria that releases dimethyl sulfide and acryloyl-CoA. *eLife* **10,** e64045 (2021).

6. Wang, S. Y. *et al.* A new dimethylsulfoniopropionate lyase of the cupin superfamily in marine bacteria. *Environ. Microbiol.* **25,** 1238-1249 (2023).

7. Wood, W. B. Host specificity of DNA produced by *Escherichia coli*: bacterial mutations affecting the restriction and modification of DNA. *J. Mol. Biol.* **16,** 118-133 (1966).

8. Studier, F. W. & Moffatt, B. A. Use of bacteriophage T7 RNA polymerase to direct selective high-level expression of cloned genes. *J. Mol. Biol*. **189,** 113-130 (1986).

9. Giaever, H. M., Styrvold, O. B., Kaasen, I. & Strøm, A. R. Biochemical and genetic characterization of osmoregulatory trehalose synthesis in *Escherichia coli*. *J Bacteriol.* **170,** 2841-2849 (1988).

10. Young, J. P. *et al*. The genome of *Rhizobium leguminosarum* has recognizable core and accessory components. *Genome Biol.* **7,** R34 (2006).

11. de Souza, M. P. & Yoch, D. C. Purification and characterization of dimethylsulfoniopropionate lyase from an *Alcaligenes*-like dimethyl sulfide-producing marine isolate. *Appl. Environ. Microbiol.* **61,** 21–26 (1995).

12. Curson, A. R. J., Sullivan, M. J., Todd, J. D. & Johnston, A. W. B. DddY, a periplasmic dimethylsulfoniopropionate lyase found in taxonomically diverse species of Proteobacteria. *ISME J.* **5,** 1191-1200 (2011).

13. Curson, A. R. J. *et al*. Dimethylsulfoniopropionate biosynthesis in marine bacteria and identification of the key gene in this process. *Nat. Microbiol.* **2,** 17009 (2017).

14. Curson, A. R. J., Rogers, R., Todd, J. D., Brearley, C. A. & Johnston, A. W. B. Molecular genetic analysis of a dimethylsulfoniopropionate lyase that liberates the climate-changing gas dimethylsulfide in several marine alpha-proteobacteria and *Rhodobacter sphaeroides*. *Environ. Microbiol*. **10,** 757-767 (2008).

15. González, J. M. *et al*. *Silicibacter pomeroyi* sp. nov. and *Roseovarius nubinhibens* sp. nov., dimethylsulfoniopropionate-demethylating bacteria from marine environments. *Int. J. Syst. Evol. Microbiol.* **53,** 1261-1269 (2003).

16. Howard, E. C. *et al*. Bacterial taxa that limit sulfur flux from the ocean. *Science.* **314,** 649–652 (2006).

17. Todd, J. D., Kirkwood, M., Newton-Payne, S. & Johnston, A. W. B. DddW, a third DMSP lyase in a model Roseobacter marine bacterium, *Ruegeria pomeroyi* DSS-3. *ISME J.* **6,** 223–226 (2012).

18. Curson, A.R.J., Fowler, E.K., Dickens, S., Johnston, A. W. B. & Todd, J. D. Multiple DMSP lyases in the γ-proteobacterium *Oceanimonas doudoroffii*. *Biogeochemistry* **110,** 109–119 (2012).

19. Todd, J. D. *et al*. Molecular dissection of bacterial acrylate catabolism - unexpected links with dimethylsulfoniopropionate catabolism and dimethyl sulfide production. *Environ Microbiol.* **12,** 327-343 (2010).

20. González, J. M., Mayer, F., Moran, M. A., Hodson, R. E. & Whitman, W. B. *Sagittula stellata* gen. nov., sp. nov., a lignin-transforming bacterium from a coastal environment. *Int. J. Syst. Bacteriol.* **47,** 773-780 (1997).

21. Johnston, A. W. B., Todd, J. D., Sun, L., Nikolaidou-Katsaridou, N., Curson, A. R. J. & Rogers, R. Molecular diversity of bacterial production of the climate-changing gas, dimethyl sulphide, a molecule that impinges on local and global symbioses. *J. Exp. Bot*. **59,** 1059-1067 (2008).

22. Cho, J. C. & Giovannoni, S. J. *Pelagibaca bermudensis* gen. nov., sp. nov., a novel marine bacterium within the Roseobacter clade in the order *Rhodobacterales*. *Int. J. Syst. Evol. Microbiol.* **56,** 855-859 (2006).

23. Todd, J. D., Curson, A. R. J, Dupont, C. L., Nicholson, P. & Johnston, A. W. B. The *dddP* gene, encoding a novel enzyme that converts dimethylsulfoniopropionate into dimethyl sulfide, is widespread in ocean metagenomes and marine bacteria and also occurs in some Ascomycete fungi. *Environ Microbiol.* **11,** 1376-1385. (2009).

24. Steinke, M., Wolfe, G.V. & Kirst, G.O. Partial characterisation of dimethylsulfoniopropionate (DMSP) lyase isozymes in 6 strains of *Emiliania huxleyi. Mar. Ecol. Prog. Ser.* **175,** 215-225 (1998).

25. Figurski, D. H. & Helinski, D. R. Replication of an origin-containing derivative of plasmid Rk2 dependent on a plasmid function provided in Trans. *PNAS.* **76,** 1648-1652 (1979).

26. Staskawicz, B., Dahlbeck, D., Keen, N. & Napoli, C. Molecular characterization of cloned avirulence genes from race 0 and race 1 of *Pseudomonas syringae* pv. *glycinea*. *J. Bacteriol.* **169,** 5789-5794 (1987).
