# Supplementary material for: DMSOP-cleaving enzymes are diverse and widely distributed in marine microorganisms
Source: Nat Microbiol. 2023 Nov 29;8(12):2326–37. doi: 10.1038/s41564-023-01526-4 (PMC10686828; doi:10.1038/s41564-023-01526-4)
Supplement: Supplementary file 2 — Reporting Summary [file 41564_2023_1526_MOESM2_ESM.pdf]

nature portfolio

Corresponding author(s): Ornella Carrion
Chun-Yang Li
Jonathan D. Todd
Yu-Zhong Zhang

Last updated by author(s): Oct 9, 2023

Reporting Summary

Nature Portfolio wishes to improve the reproducibility of the work that we publish. This form provides structure for consistency and transparency in reporting. For further information on Nature Portfolio policies, see our Editorial Policies and the Editorial Policy Checklist.

Statistics

For all statistical analyses, confirm that the following items are present in the figure legend, table legend, main text, or Methods section.

n/a	Confirmed	
☐	☒	The exact sample size (*n*) for each experimental group/condition, given as a discrete number and unit of measurement
☐	☒	A statement on whether measurements were taken from distinct samples or whether the same sample was measured repeatedly
☐	☒	The statistical test(s) used AND whether they are one- or two-sided<br>*Only common tests should be described solely by name; describe more complex techniques in the Methods section.*
☒	☐	A description of all covariates tested
☒	☐	A description of any assumptions or corrections, such as tests of normality and adjustment for multiple comparisons
☐	☒	A full description of the statistical parameters including central tendency (e.g. means) or other basic estimates (e.g. regression coefficient) AND variation (e.g. standard deviation) or associated estimates of uncertainty (e.g. confidence intervals)
☐	☒	For null hypothesis testing, the test statistic (e.g. *F*, *t*, *r*) with confidence intervals, effect sizes, degrees of freedom and *P* value noted<br>*Give P values as exact values whenever suitable.*
☒	☐	For Bayesian analysis, information on the choice of priors and Markov chain Monte Carlo settings
☒	☐	For hierarchical and complex designs, identification of the appropriate level for tests and full reporting of outcomes
☒	☐	Estimates of effect sizes (e.g. Cohen's *d*, Pearson's *r*), indicating how they were calculated

Our web collection on statistics for biologists contains articles on many of the points above.

Software and code

Policy information about availability of computer code

Data collection	X-ray diffraction data were collected on the BL17U1, BL18U1 and BL19U1 beamlines at the Shanghai Synchrotron Radiation Facility
Data analysis	GC-HRMS data was processed with Thermo Xcalibur software v4.5.445.18. Distribution of genes of interest in Tara Oceans datasets was analysed using Ocean Gene Atlas v2.0. X-ray diffraction data was processed using HLK3000 v715. Crystal structures were determined and refined using CCP4 phaser v6.5, WinCoot v0.8.1 and Phenix v1.16-3549 and images processed with PyMOL v1.6.0.0. DMSOP chemical structure was obtained with Chem Draw and Chem Draw 3D v17.1.0.105. Molecular docking was performed using the Docking tool in the Zcloud platform (https://cloud.zelixir.com).

For manuscripts utilizing custom algorithms or software that are central to the research but not yet described in published literature, software must be made available to editors and reviewers. We strongly encourage code deposition in a community repository (e.g. GitHub). See the Nature Portfolio guidelines for submitting code & software for further information.

Data

Policy information about availability of data

All manuscripts must include a data availability statement. This statement should provide the following information, where applicable:

- Accession codes, unique identifiers, or web links for publicly available datasets
- A description of any restrictions on data availability
- For clinical datasets or third party data, please ensure that the statement adheres to our policy

The structure of DddK-DMSOP and DddY-DMSOP complexes generated in this study are publicly available from the Protein Data Bank (PDB) under accession numbers 8HLF and 8HLE.

Human research participants

Policy information about studies involving human research participants and Sex and Gender in Research.

Reporting on sex and gender	N/A
Population characteristics	N/A
Recruitment	N/A
Ethics oversight	N/A

Note that full information on the approval of the study protocol must also be provided in the manuscript.

Field-specific reporting

Please select the one below that is the best fit for your research. If you are not sure, read the appropriate sections before making your selection.

☒ Life sciences ☐ Behavioural & social sciences ☐ Ecological, evolutionary & environmental sciences

For a reference copy of the document with all sections, see nature.com/documents/nr-reporting-summary-flat.pdf

Life sciences study design

All studies must disclose on these points even when the disclosure is negative.

Sample size	No sample calculations were performed. Experiments with saltmarsh sediments were done with three biological replicates as is standard practice for environmental sampling. All experiments with bacteria, algae, fungi and purified proteins reported were performed with biological replicates. The use of three biological replicates is standard accepted practice for microbiology. See methods section.
Data exclusions	No data was excluded from this study.
Replication	All experiments were repeated three times as completely independent experiments with equivalent results, except for experiments with saltmarsh sediments where no replication was done.
Randomization	No randomization was done as experiments were done with model organisms.
Blinding	Blinding is not relevant to this study as experiments were done with model organisms.

Reporting for specific materials, systems and methods

We require information from authors about some types of materials, experimental systems and methods used in many studies. Here, indicate whether each material, system or method listed is relevant to your study. If you are not sure if a list item applies to your research, read the appropriate section before selecting a response.

Materials & experimental systems

n/a	Involved in the study
☒	☐ Antibodies
☒	☐ Eukaryotic cell lines
☒	☐ Palaeontology and archaeology
☒	☐ Animals and other organisms
☒	☐ Clinical data
☒	☐ Dual use research of concern

Methods

n/a	Involved in the study
☒	☐ ChIP-seq
☒	☐ Flow cytometry
☒	☐ MRI-based neuroimaging
